# Prevalence of cardiovascular risk factors in active tuberculosis in Africa: a systematic review and meta-analysis

**DOI:** 10.1038/s41598-022-20833-0

**Published:** 2022-09-29

**Authors:** Joseph Baruch Baluku, Olum Ronald, Peace Bagasha, Emmy Okello, Felix Bongomin

**Affiliations:** 1 Division of Pulmonology, Kiruddu National Referral Hospital, PO Box 26343, Kampala, Uganda; 2 Makerere University Lung Institute, Kampala, Uganda; 3 Department of Internal Medicine, School of Medicine, Makerere University College of Health Sciences, Kampala, Uganda; 4 Uganda Heart Institute, Kampala, Uganda; 5 Department of Medical Microbiology and Immunology, Faculty of Medicine, Gulu University, Gulu, Uganda

**Keywords:** Cardiology, Risk factors

## Abstract

People with tuberculosis (TB) are at risk of major adverse cardiovascular events. We estimated the prevalence of cardiovascular risk (CVR) factors among people with active TB in Africa. This was a systematic review and meta-analysis of studies from Africa. We searched EMBASE, MEDLINE through PubMed, Web of Science, the Cochrane Central Register of Controlled Trials, mRCTs, Clinical trials.gov, and International Clinical Trials Registry Platform from inception to 31st December 2021. Among 110 eligible studies, 79 (238,316 participants) were included in the meta-analysis for smoking, 67 (52,793 participants) for current alcohol use, 30 (31,450 participants) for hazardous alcohol use, 51 (37,879 participants) for diabetes mellitus (DM), 19 (18,211 participants) for hypertension and 18 (13,910 participants) for obesity. The pooled prevalence was 26.0% (95% confidence interval 22.0–29.0) for smoking, 30.0% (25.0–35.0) for any current alcohol use, 21.0% (17.0–26.0) for hazardous alcohol use, 14.0% (9.0–18.0) for hypertension, 7.0% (6.0–9.0) for DM, and 4.0% (2.0–5.0) for obesity. Cost-effective strategies are needed to screen for CVR factors among people with active TB in Africa.

## Introduction

The global burden of cardiovascular disease (CVD) has nearly doubled in the last two decades from 271 to 523 million cases between 1990 and 2019^1^. In Africa, the burden of CVD has increased due to the rise in traditional cardiovascular risk (CVR) factors^2^. CVD accounts for 13% of all deaths and 37% of non-communicable disease-related deaths in sub-Saharan Africa^3^. At the same time, the region is still grappling with a high incidence of infectious diseases such as HIV and tuberculosis (TB). Accordingly, a convergence of cardiovascular and infectious diseases is observed in African countries^4^.

Africa contributed 25% of the global TB cases in 2019^5^. The interaction between TB and CVD is complex. Latent TB infection increases the risk of hypertension independent of body mass index, HIV infection and serum cholesterol^6^. *Mycobacterium tuberculosis* can also directly cause myocarditis, aortitis and pericarditis^7^. As such, people with active TB have a 51% higher risk for major adverse cardiovascular events than controls^8^. Several observational studies show that people with active TB are at a higher risk of ischemic stroke^9^, myocardial infarction^10^, peripheral artery disease^11^, deep venous thrombosis, pulmonary embolism and venous thromboembolism^12^. There is need to characterise the burden of CVD risk factors among people with active TB because CVD risk factors can synergistically increase the risk for CVD-related mortality among these people. Moreover, CVD accounts for 20% of deaths among survivors of TB after TB treatment completion^13^. On the one hand, CVR factors such as smoking^14,15^, alcohol use^16,17^ and diabetes mellitus (DM)^18,19^ are risk factors for TB infection and poor TB outcomes. On the other hand, obesity is protective against TB infection and adverse outcomes^20,21^.

While the prevalence of DM in TB in Africa is estimated at 7.7–9.0%^22,23^, the burden of other CVR factors in active TB in Africa is not well characterised. Determining the burden of CVR factors in active TB in Africa informs the need, if any, of integrating CVR scoring and risk modification interventions in routine TB care. We, therefore, determined the prevalence of CVR factors among people with active TB in Africa.

## Methods

### Search strategy and selection criteria

We performed a systematic review and meta-analysis of studies reporting the prevalence of CVR factors among people with active TB in Africa. The CVR factors of focus were hypertension, DM, dyslipidaemia (lipid abnormalities), obesity, physical inactivity, alcohol use and smoking. The study was conducted according to the Preferred Reporting Items for Systematic Reviews and Meta-Analyses (PRISMA) guidelines^24^. The study protocol was developed following the PRISMA-P guidelines^25^ and registered on PROSPERO (registration number: CRD42021245395).

We searched for all studies published from inception to 31st December 2020 and later updated the search to 31st December 2021 (Supplementary Material Table 10). The following databases were comprehensively searched: EMBASE, MEDLINE through PubMed, Web of Science, the Cochrane Central Register of Controlled Trials (CENTRAL), mRCTs, Clinical trials.gov, and International Clinical Trials Registry Platform (ICTRP). The following medical subject headings (MeSH) terms were used: “prevalence” OR “incidence” OR “burden” AND “hypertension”, “diabetes”, “pre-diabetes”, “cardiovascular risk factors”, “metabolic syndrome” “hyperlipidaemia”, “dyslipidemia”, “cholesterol”, “hypercholesterolemia”, “low density lipoprotein”, “triglycerides”, “alcohol”, “smoking”, “cigarette”, “obesity”, “overweight”, “physical inactivity”, AND ”tuberculosis”, “TB”, “PTB”, AND “Africa” OR the individual names of the African countries. The search was limited to studies published in English and French; the predominant languages used in Africa.

We included prospective, cross-sectional, retrospective, and interventional studies reporting the prevalence (or for which the proportion could be calculated) of any of hypertension, DM, lipid abnormalities, obesity, physical inactivity, alcohol use and smoking among people with active TB in Africa. We excluded case reports, case series with subjects less than 10, opinion papers, qualitative research, letters to the editor, comments, conference proceedings, policy papers, reviews and meta-analyses, study protocols without baseline data, and animal studies.

After the database search, duplicates were removed using the Healthcare Databases Advanced Search program (National Institute for Health and Care Excellence, UK). Thereafter, articles were reviewed by title and abstract by two reviewers (JBB and RO) to remove articles that are unrelated to the study question. The full text of the articles that passed this initial screen were then retrieved and assessed by two investigators independently (JBB and FB) (Supplementary Table 7). Any disagreements were resolved by consensus. Data were extracted by three independent reviewers (JBB, FB and RO) with Microsoft Excel^®^ using a data abstraction form. The form captured study design, year of publication, number of participants, country where the study was conducted, type of TB by drug resistance profile, criteria used for classifying individuals as having a given CVR factor and reported prevalence (or proportion) of the CVR factors. Any variation in the extracted data by the reviewers was discussed and resolved by consensus.

The primary study outcome was the prevalence of any current alcohol use, smoking, hypertension, DM, lipid abnormalities, obesity and physical inactivity among people with active TB in Africa. Hypertension was defined as a systolic blood pressure (SBP) ≥ 140 mmHg and/or diastolic blood pressure (DBP) ≥ 90 mmHg or use of anti-hypertension medication or “known patient with hypertension”. Obesity was defined as a body mass index (BMI) of ≥ 30 kg per m^2^. DM was defined as glycated haemoglobin (HbA1c) level ≥ 6.5% or fasting blood sugar (FBS) ≥ 126 mg/dl or 2 h plasma glucose of ≥ 200 mg/dl after oral glucose tolerance test or random blood sugar (RBS) ≥ 11.1 mmol/l with symptoms of DM or use of DM medication. Hazardous alcohol use was operationally defined a posteriori as any of: daily use of alcohol, consumption of alcohol on ≥ 3 days of the week, studies describing users as "chronic drinker”, “misusing alcohol”, or reporting a prevalence of “alcoholism", alcohol dependence measured by the Mini International Neuropsychiatric Interview, Alcohol Use Disorders Identification Test (AUDIT) score of ≥ 8^26^, and the cut down, anger, guilt and eye-opener (CAGE) questionnaire score of ≥ 2^27^. Any history of smoking was considered in estimating the prevalence of smoking.

Using a tool by Hoy et al.^28^ for assessing risk of bias in prevalence studies, two independent reviewers (RO and FB) evaluated the quality of the studies for risk of bias and risk was graded as low (> 8), moderate (5–8) and high (< 5) (Supplementary Table 6).

### Statistical analysis

Data were analysed using STATA 16.0 (StataCorp LLC, Texas, USA). Heterogeneity of the data was assessed using the Q statistic and *I*^*2*^ index and the corresponding p-value. Heterogeneity was considered as low (*I*^2^ = 0–25%), moderate (*I*^2^ = 26–50%), or high (*I*^2^ > 50%). Depending on the heterogeneity of the data, random-effect (for I^2^ ≥ 50%) or fixed-effect (for I^2^ < 50%) models were used to determine the pooled prevalence of a given CVR factor presented as a proportion and the corresponding 95% confidence interval. Forest plots were used to present the results of the meta-analysis. Publication bias was assessed visually using funnel plots and statistically using Egger’s regression test. We further determined whether the observed asymmetry was due to publication bias via enhanced-contour funnel plots after the trim-and-fill method. A sensitivity analysis was performed for the prevalence of a given CVR by region, among studies within the funnel plot (Supplementary Material: Figs. 1–6), among people with drug resistant TB (DRTB), and risk of bias of the study. We further performed meta-regression analyses to assess sources of heterogeneity (Supplementary Material: Table 8). A two-tailed p < 0.05 was considered statistically significant.

## Results

### Study characteristics

We identified 110 eligible studies (Supplementary Fig. 7). The mean (standard deviation) risk of bias score was 7.9 (1.4). Majority of studies (63.6%, 70/110) had a score of ≥ 8. The study summary statistics are shown in Table 1. Among these studies, 79 (238,316 participants) were included in the meta-analysis for smoking, 67 (52,793 participants) for current alcohol use, 30 (31,450 participants) for hazardous alcohol use, 51 (37,879 participants) for DM, 19 (18,211 participants) for hypertension and 18 (13,910 participants) for obesity. Two studies (4320 participants)^29,30^ reported on lipid abnormalities and another two studies^31,32^ reported data on physical inactivity (2247 participants). Therefore, meta-analyses for lipid abnormalities and physical inactivity were not performed. Supplementary Tables 1–5 show characteristics of studies included in the meta-analyses for each CVR factor.

**Table 1:** Summary Statistics for the prevalence of cardiovascular risk factors among people with active TB in Africa.

Cardiovascular risk factor	No. studies	No. participants	Pooled prevalence (%)	95% confidence interval (CI) (%)	Heterogeneity (I^2^) (%)	p_heterogeneity_	p_Egger’s_
**Smoking**							
Overall	79	238,316	26	22–29	99.6	< 0.001	0.005
Studies in funnel plot	15	199,641	22	21.8–22.2	4.01	< 0.001	0.235
DRTB only	10	3751	27	16–37	98.4	< 0.001	
Southern Africa	35	219,397	30	25–36	99.7	< 0.001	
West Africa	16	10,294	18	10–25	99.3	< 0.001	
East Africa	23	7823	21	16–26	98.3	< 0.001	
Northern Africa	4	507	45	29–61	92.7	< 0.001	
**Current alcohol use**							
Overall	67	52,793	30	25–35	99.5	< 0.001	0.161
Studies in funnel plot	15	4382	28	26–29	0.0	< 0.001	0.421
DRTB only	10	3356	27	16–38	98.6	< 0.001	
Southern Africa	28	37,327	29	21–36	99.7	< 0.001	
West Africa	12	7502	29	15–43	99.6	< 0.001	
East Africa	24	7596	32	24–40	98.8	< 0.001	
**Hazardous alcohol use**							
Overall	30	31,450	21	17–26	99.1	< 0.001	0.001
Studies in funnel plot	5	3978	21	20–22	35.1	< 0.001	0.535
DRTB only	3	733	15	2–28	97.1	< 0.001	
Southern Africa	17	26,586	21	16–27	99.3	< 0.001	
West Africa	3	1703	13	2–25	96.8	< 0.001	
East Africa	8	2793	22	11–33	98.6	< 0.001	
**Diabetes**							
Overall	51	37,879	7	6–9	98.5	< 0.001	< 0.001
Studies in funnel plot	18	10,922	4	4–4	0.0	< 0.001	0.787
DRTB only	9	3587	7	2–12	97.6	< 0.001	
Southern Africa	19	19,029	6	4–7	94.2	< 0.001	
West Africa	11	6914	6	3–10	96.5	< 0.001	
East Africa	19	11,702	9	5–13	99.1	< 0.001	
**Hypertension**							
Overall	19	18,211	14	9–18	99.1	< 0.001	0.022
Studies in funnel plot	7	6001	8	7.8–9.2	0.0	0.470	0.203
DRTB only	6	2008	13	4–22	97.3	< 0.001	
Southern Africa	11	14,601	14	8–19	99.3	< 0.001	
West Africa	4	2044	6	1–11	94.3	< 0.001	
East Africa	4	1566	22	12–33	96.5	< 0.001	
**Obesity**							
Overall	18	13,910	4	2–5	97.0	< 0.001	0.002
Studies in funnel plot	8	1026	1.5	0.9–2.1	34.8	0.150	0.010
Southern Africa	10	9661	5	3–7	90.4	< 0.001	
West Africa	4	1962	5	1–10	98.0	< 0.001	
East Africa	4	2287	2	0–3	81.9	< 0.001	

### Prevalence of smoking

**Figure 1:**
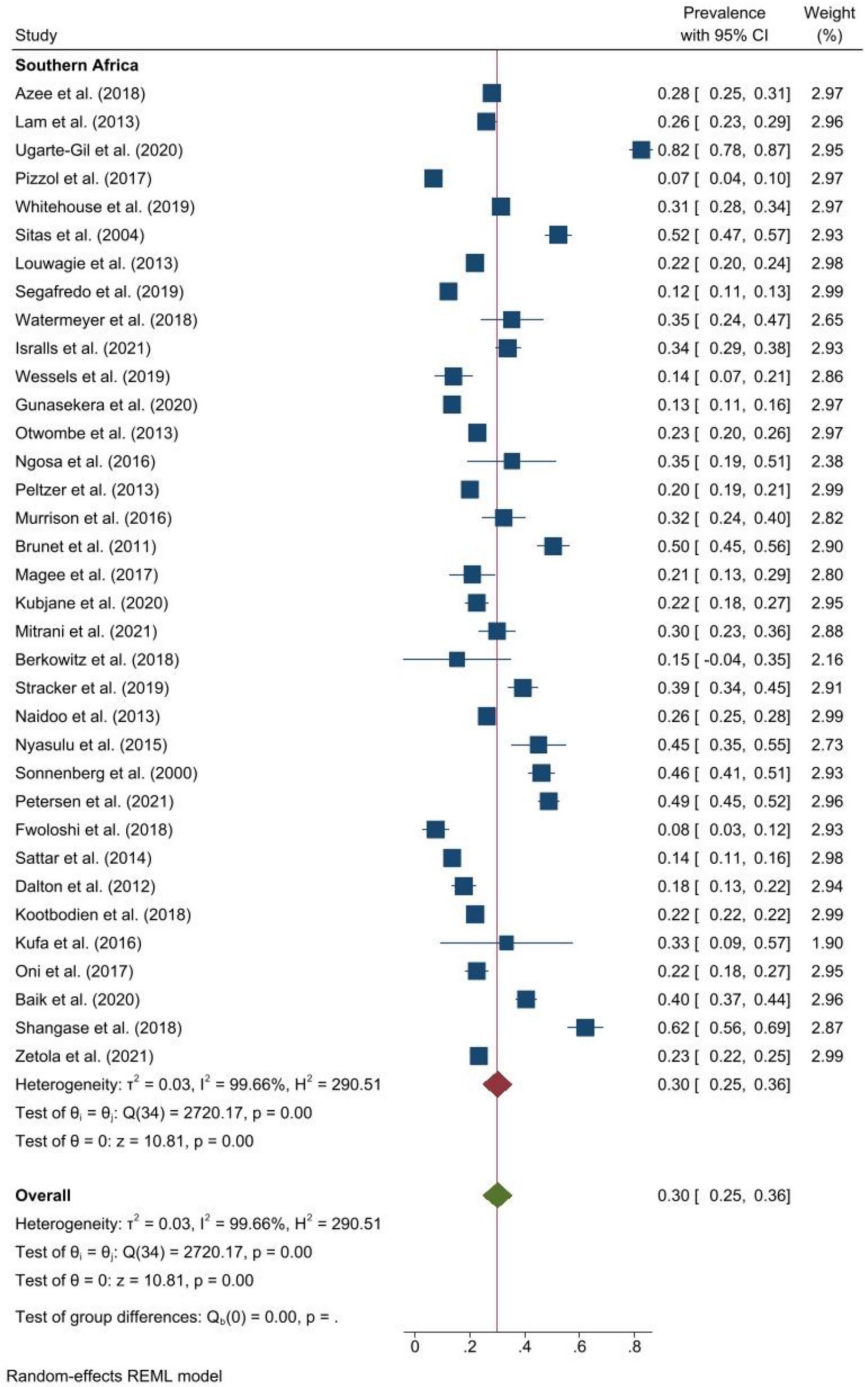
Forest plot showing the pooled prevalence of smoking among people with active TB in Southern Africa.

**Figure 2:**
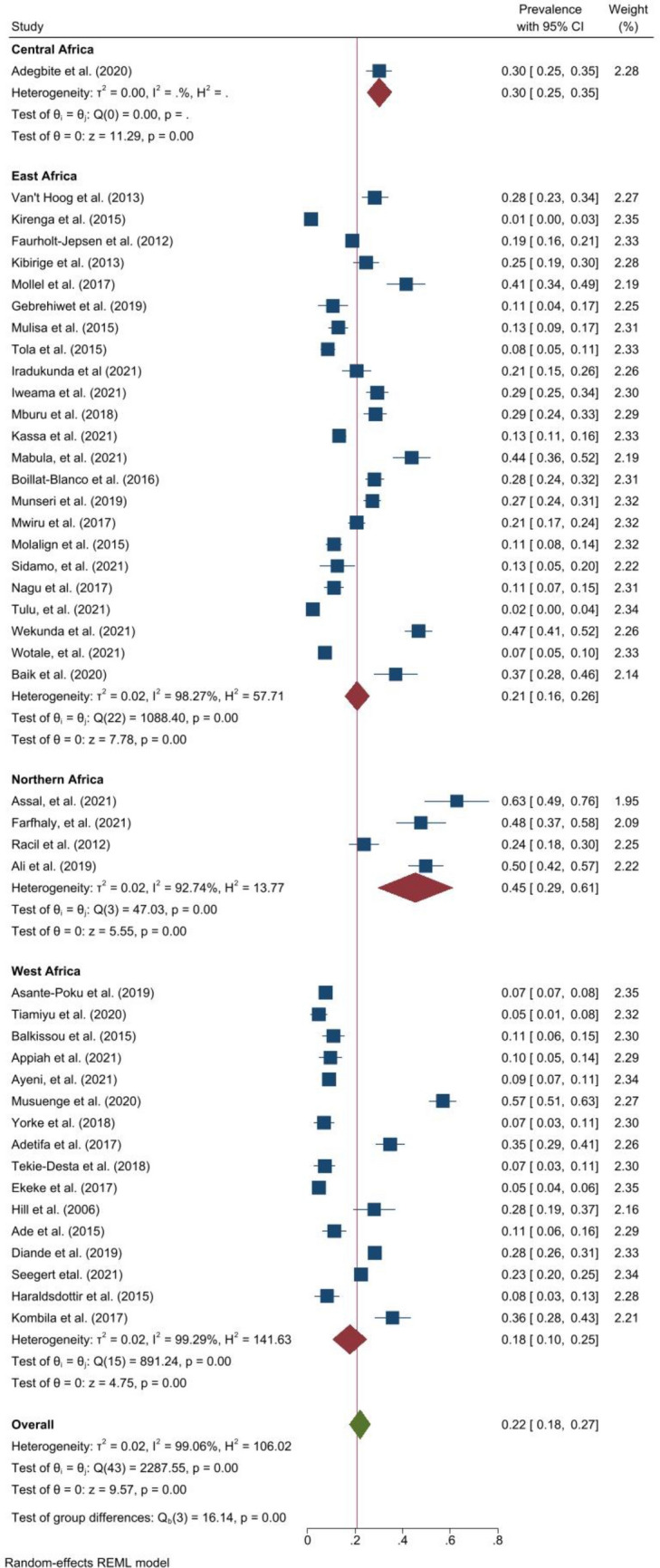
Forest plot showing the pooled prevalence of smoking among people with active TB in East, West, Northern and Central Africa.

The pooled prevalence of any history of smoking was 26.0% (95% CI 22.0–29.0, *I*^*2*^ = 99.6%, p < 0.001) and ranged from 1.4% in Uganda^33^ to 82.5% in South Africa^34^. Among people with DRTB, the pooled prevalence of smoking was 27.0% (95% CI 16.0–37.0, *I*^*2*^ = 98.4%, p < 0.001)^35–44^. The pooled prevalence of any history of smoking was 45.0% (95% CI 29.0–61.0, *I*^*2*^ = 92.7%, p < 0.001) in Northern Africa, 30.0% (95% CI 25.0–36.0, *I*^*2*^ = 99.7%, p < 0.001) in Southern Africa (Fig. 1), 21.0% (95% CI 16.0–26.0, *I*^*2*^ = 98.3%, p < 0.001) in East Africa, and 18.0% (95% CI 10.0–25.0, *I*^*2*^ = 99.3%, p < 0.001) in West Africa. One study in Central Africa reported the prevalence at 30.0%^45^. Figure 2 shows the forest plot for the prevalence of smoking in Western, Eastern, and Northern Africa. Among studies within the funnel plot, the pooled prevalence of smoking was 22.0% (95% CI 22.0–22.0, *I*^*2*^ = 4.0%, p < 0.001). Similarly, among studies with low risk of bias, the pooled prevalence was 22.0% (95% CI 14.0–31.0, *I*^*2*^ = 99.8%, p < 0.001) and 27.0% (95% CI 23.0–31.0, *I*^*2*^ = 99.1%, p < 0.001) among studies with moderate risk of bias.

### Prevalence of alcohol use

**Figure 3:**
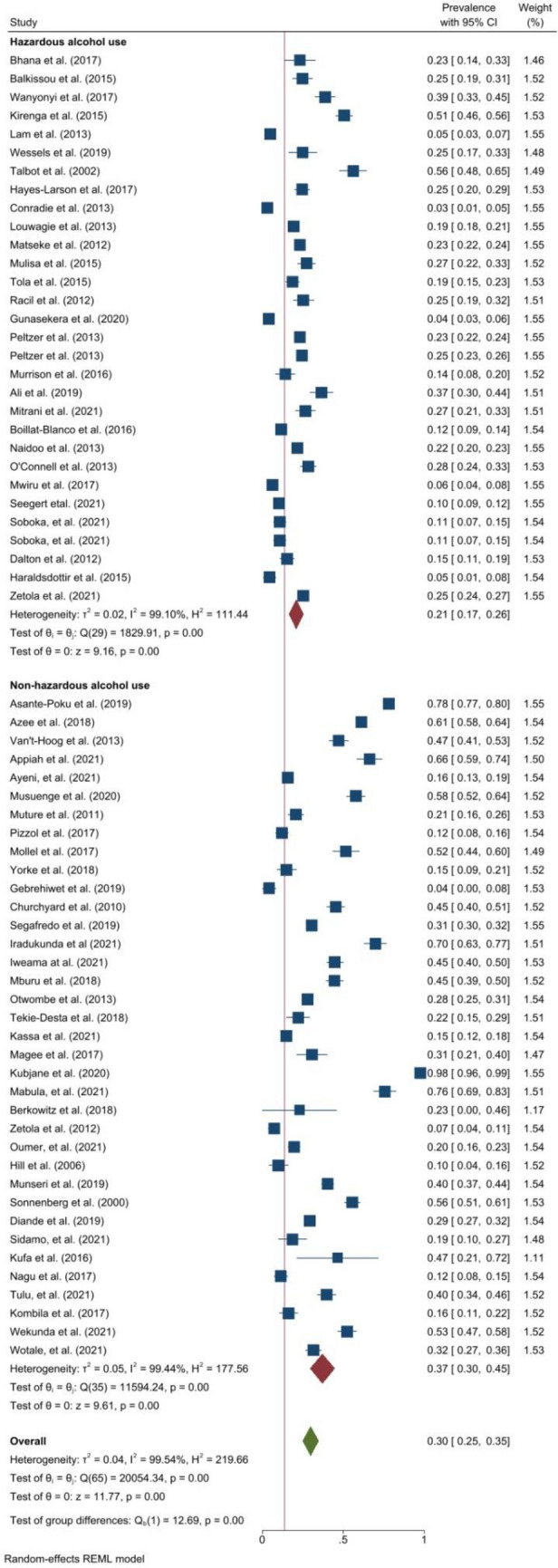
Forest plot showing the prevalence of alcohol use among people with active TB in Africa.

The pooled prevalence of any current alcohol use (Fig. 3) was 30.0% (25.0–35.0, *I*^*2*^ = 99.5%, p < 0.001). The prevalence ranged from 3.3% in South Africa^46^ to 97.8% in another study from South Africa^47^. In DRTB, the pooled prevalence of any current use was 27.0% (95% CI 16.0–38.0, *I*^*2*^ = 98.6%, p < 0.001)^35,36,38,40,42,43,46,48–50^. Across the regions, the pooled prevalence of any current alcohol use was highest in East Africa but similar in other regions; that is, 32.0% (95% CI 24.0–40.0, *I*^*2*^ = 98.8%, p < 0.001) in East Africa, 29.0% (95% CI 21.0–36.0, *I*^*2*^ = 99.7%, p < 0.001) in Southern Africa, and 29.0% (95% CI 15.0–43.0, *I*^*2*^ = 99.6%, p < 0.001) in West Africa. In Northern Africa, two studies reported a prevalence of 25.0–37.0%^51,52^. For studies within the funnel plot, the pooled prevalence of any current was 28.0% (95% CI 26.0–29.0, *I*^*2*^ = 0.0%, p < 0.001). Among studies with low risk of bias, the pooled prevalence was 29.0% (95% CI 20.0–38.0, *I*^*2*^ = 99.8%, p < 0.001) and 30.0% (95% CI 24.0–36.0, *I*^*2*^ = 99.2%, p < 0.001) among studies with moderate risk of bias.

### Prevalence of hazardous alcohol use

The pooled prevalence of hazardous alcohol use was 21.0% (95% CI 17.0–26.0, *I*^*2*^ = 99.1%, p < 0.001). The estimates were similar among studies within the funnel plot, low and moderate risk of bias. That is, 21.0% (95% CI 20.0–22.0, *I*^*2*^ = 35.1%, p < 0.001) among studies within the funnel plot, 21.0% (95% CI 16.0–25.0, *I*^*2*^ = 98.3%, p < 0.001) in studies with low risk of bias, and 21.0% (95% CI 14.0–29.0, *I*^*2*^ = 99.2%, p < 0.001) in studies with moderate risk of bias. Only three studies^36,46,48^ reported the prevalence of hazardous alcohol use in DRTB with a pooled prevalence of 15.0% (95% CI 2.0–28.0, *I*^*2*^ = 97.1%, p = 0.03).

### Prevalence of DM

**Figure 4:**
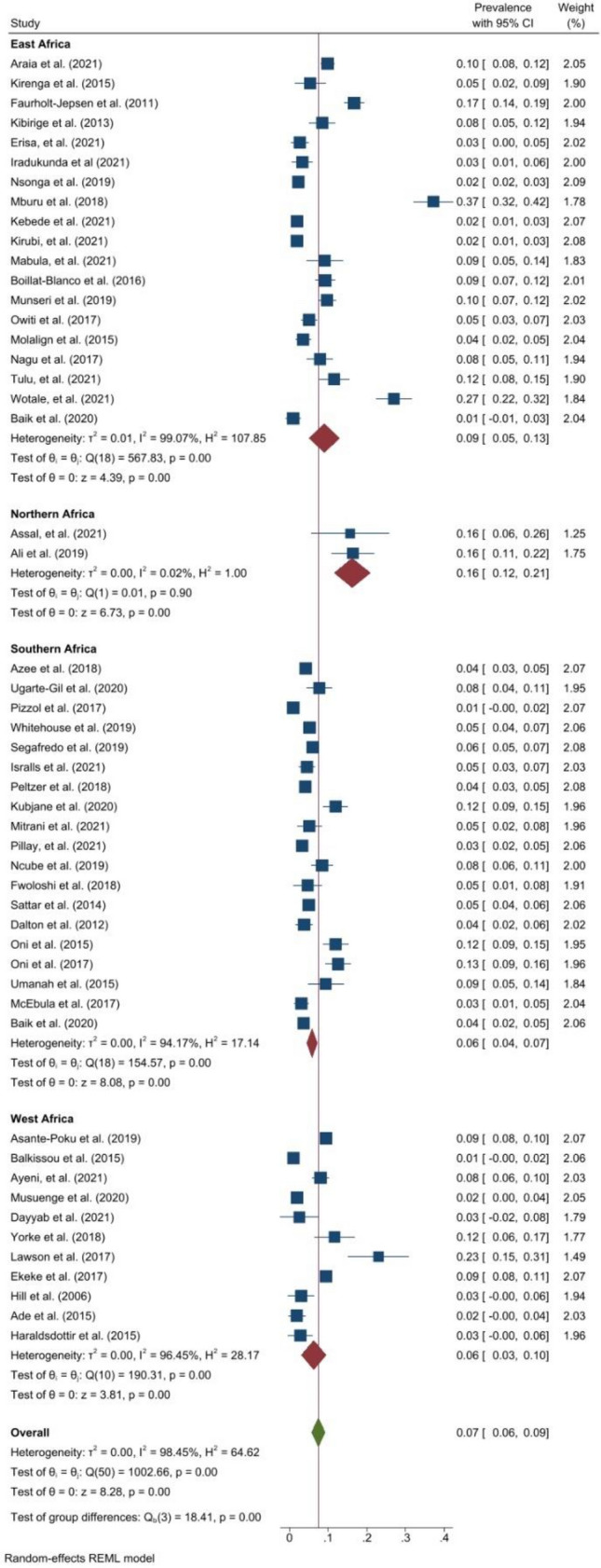
Forest plot showing the prevalence of DM among people with active TB in Africa.

The pooled prevalence of DM (Fig. 4) was 7.0% (95% CI 6.0–9.0, *I*^*2*^ = 98.5%, p < 0.001). The prevalence of DM ranged from 1.0% in studies from Uganda^53^, Mozambique^54^ and Cameroon^55^ to 37.2% in Kenya^56^. However, Mburu et al.^56^ used HbA1c of > 6.0% as the cut-off for defining DM in Kenya. Among patients with DRTB, the pooled prevalence was also 7.0% (95% CI 2.0–12.0, *I*^*2*^ = 97.6%, p < 0.001)^35–37,39,42,44,48,57^. The pooled prevalence of DM was highest in East Africa at 9.0% (95% CI 5.0–13.0, *I*^*2*^ = 99.1%, p < 0.001) and was similar in West Africa (6.0% (95% CI 3.0–10.0, *I*^*2*^ = 96.5%, p < 0.001)) and Southern Africa at 6.0% (4.0–7.0, *I*^*2*^ = 94.2%, p < 0.001). In Northern Africa, the prevalence of DM was reported by two studies at 16.5%^51^ and 15.7%^58^. The pooled prevalence of DM was 4.0% (95% CI 4.0–4.0, *I*^*2*^ = 0.0%, p < 0.001) among studies within the funnel plot. Among studies with low risk of bias, the pooled prevalence was 8.0% (95% CI 6.0–10.0, *I*^*2*^ = 96.8%, p < 0.001) and 7.0% (95% CI 5.0–10.0, *I*^*2*^ = 98.5%, p < 0.001) among studies with moderate risk of bias.

### Prevalence of hypertension

**Figure 5:**
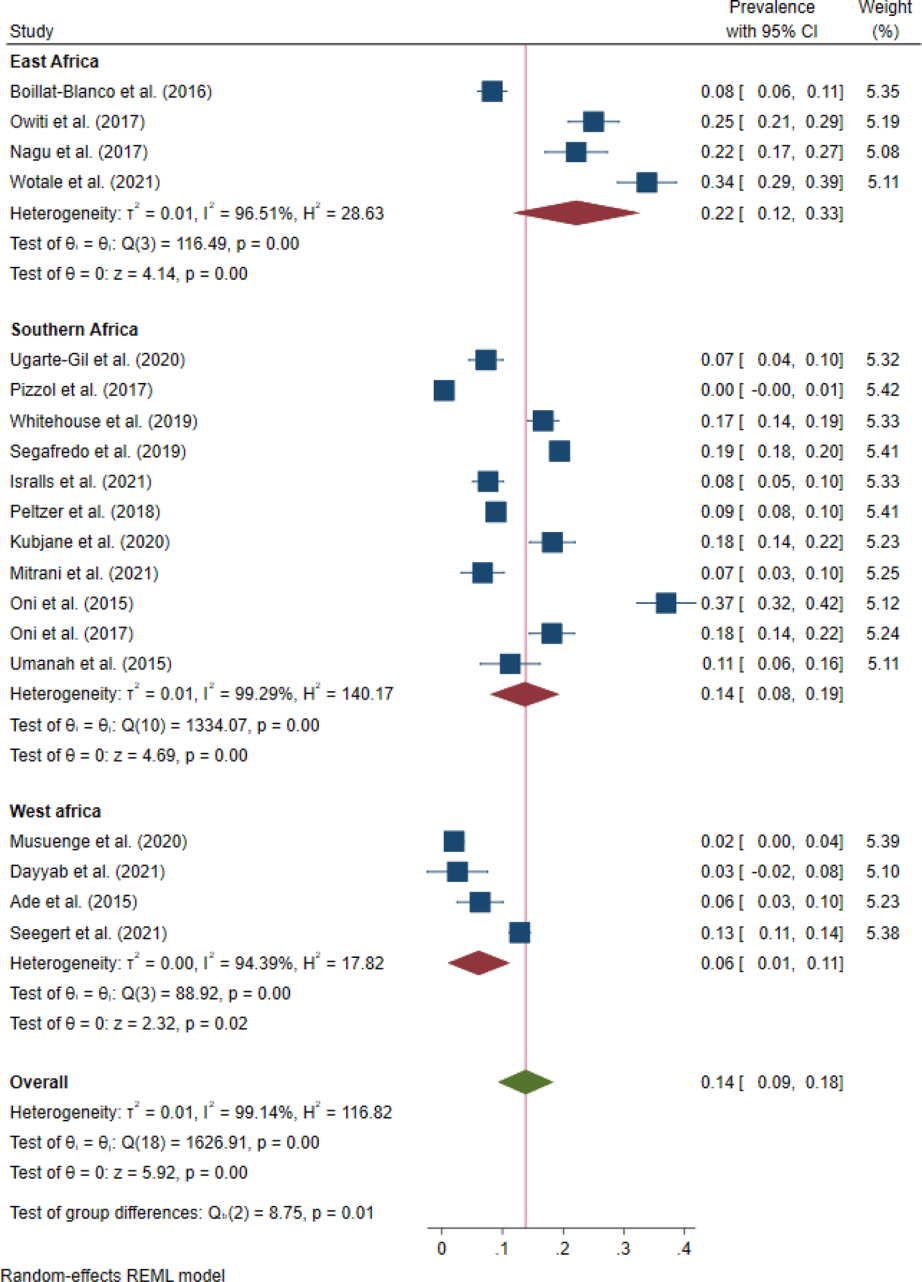
Forest plot showing prevalence of hypertension among people with active TB in Africa.

The pooled prevalence of hypertension (Fig. 5) was 14.0% (95% CI 9.0–18.0, *I*^*2*^ = 99.1%, p < 0.001). The prevalence of hypertension ranged from 0.3% in Mozambique^54^ to 37.0% in South Africa^59^. The prevalence in DRTB was 13.0% (95% CI 4.0–22.0, *I*^2^ = 97.3%, p < 0.001) in six studies^37,42,44,48,57,60^. East Africa had the highest pooled prevalence of hypertension at 22.0% (95% CI 12.0–33.0, *I*^2^ = 96.5%, p < 0.001) followed by Southern Africa at 14.0% (95% CI 8.0–19.0, *I*^2^ = 99.3%, p < 0.001) and West Africa at 6.0% (95% CI 1.0–11.0, *I*^2^ = 94.4%, p < 0.001). Among studies within the funnel plot, the pooled prevalence of hypertension was 9.0% (95% CI 8.0–9.0, *I*^2^ = 0.0%, p < 0.001). Among studies with low risk of bias, the pooled prevalence was 14.0% (95% CI 10.0–18.0, *I*^*2*^ = 98.9%, p < 0.001) and 13.0% (95% CI 4.0–23.0, *I*^*2*^ = 98.5%, p < 0.001) among studies with moderate risk of bias.

### Prevalence of obesity

**Figure 6:**
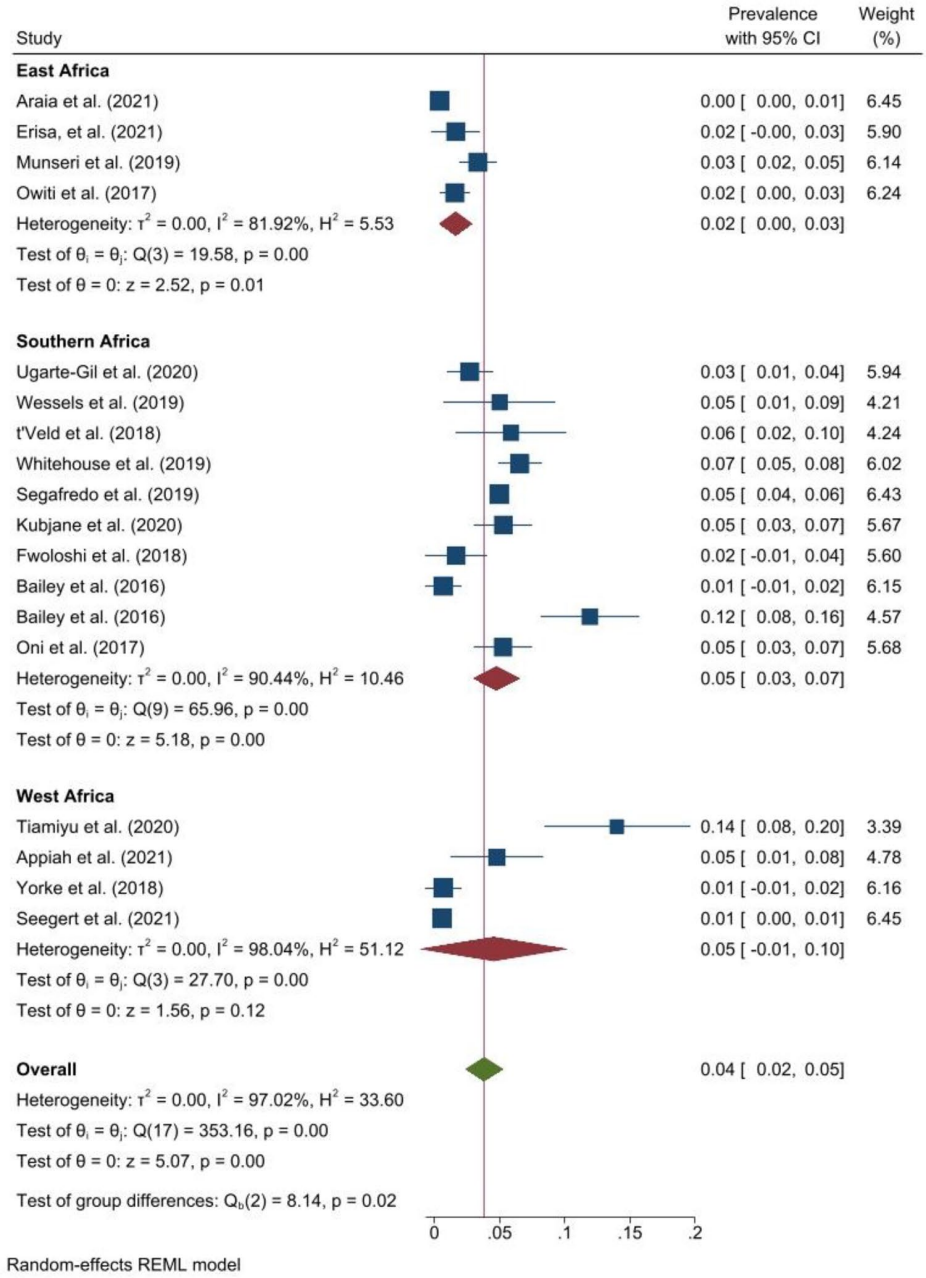
Forest plot showing prevalence of obesity among people with active TB in Africa.

The pooled prevalence of obesity was 4.0% (95% CI 2.0–5.0, *I*^2^ = 97.0%, p < 0.001) (Fig. 6). The prevalence ranged from 0.4% in Eritrea^61^ to 14.0% in Nigeria^62^. One study reported a prevalence of 7.0% (95% CI 5.0–8.0) among people with DRTB^37^. The pooled prevalence of obesity in Southern Africa was 5.0% (95% CI 3.0–7.0, *I*^2^ = 90.4%, p < 0.001), 5.0% (95% CI 1.0–10.0, *I*^2^ = 98.0, p < 0.001 ) in West Africa and 2.0% (95% CI 0.0–3.0, *I*^2^ = 81.9%, p < 0.001) in East Africa. The pooled prevalence of obesity was 2.0% (95% CI 1.0–2.0, *I*^2^ = 34.8%, p = 0.150) among studies within the funnel plot. Among studies with low risk of bias, the pooled prevalence was 5.0% (95% CI 3.0–7.0, *I*^*2*^ = 97.6%, p < 0.001) and 2.0% (95% CI 1.0–3.0, *I*^*2*^ = 77.3%, p < 0.001) among studies with moderate risk of bias.

### Prevalence of lipid abnormalities and physical inactivity

With regards to lipid abnormalities, 67% of people with TB had low high density lipoproteins in Nigeria^29^ while only 1.7% self-reported any lipid abnormalities in South Africa^30^. Physical inactivity, defined as a sedentary occupation and “no physical exercise”, was reported in 49.8% of people with TB in Nigeria and 69.9% in Tanzania, respectively^31,32^. In the study from Nigeria, 32.9% had moderate activity and 17.3% had occupations associated with vigorous activity.

### Heterogeneity and publication bias

There was high heterogeneity (*I*^2^ > 50%) in the estimation of the pooled prevalence of all CVR factors (Table 1) except for studies within the funnel plots. In the meta-regression analysis (Supplementary Material Table 8), there were significant differences in the prevalence of hypertension and smoking across the regions of Africa. Current alcohol use also varied between studies that reported hazardous and non-hazardous use. Visually, the funnel plots (Supplementary Fig. 8) were asymmetrical, suggesting overall publication bias for the studies included in the meta-analyses. This was confirmed by Egger’s test for smoking (p = 0.005), hazardous alcohol use (p = 0.001), DM (p < 0.001), hypertension (p = 0.022) and obesity (p = 0.002) but not current alcohol use (p = 0.161). The enhanced-contour funnel plots after the trim-and-fill method further demonstrated publication bias in the estimates for the prevalence of obesity and current alcohol use (Supplementary Material Fig. 9 and Table 9).

## Discussion

In this systematic review and meta-analysis, we have provided the first comprehensive estimates for the burden of traditional CVR factors among people with active TB in Africa. The prevalence was 26.0% for smoking, 30.0% for any current alcohol use, 21.0% for hazardous alcohol use, 14.0% for hypertension, 7.0% for DM, and 4.0% for obesity. There was substantial heterogeneity in these estimates, and this could be explained by variations in the prevalence by regions of Africa and publication bias. Accordingly, East Africa had the highest prevalence of hypertension and DM while Northern Africa had the highest prevalence of smoking. People with active DRTB had comparable estimates in the prevalence of all CVR factors.

The prevalence of smoking and DM in this study is higher than what is reported in the general African population for smoking (8.3%)^63^ and undiagnosed DM (3.9–5.4%)^64^. This is possibly because smoking^65^ and DM^66^ are well established risk factors for active TB. Moreover, smoking interacts with DM to synergistically increase the risk for TB^67^. The high prevalence of smoking and DM in active TB is concerning because both increase the risk of adverse TB treatment outcomes^14,68^. It was interesting to observe similar prevalence of DM in DRTB even when the criteria for the diagnosis of DM in DRTB was unknown in four of the five studies included in the sub-analysis for DM^35,36,39,57^. Therefore, although the true burden of DM in DRTB in Africa remains largely unknown, it is likely to be similar to that among people with susceptible TB. Our findings suggest a need for cost effective strategies for screening for DM and smoking among people with active TB in Africa. The prevalence of DM in active TB in our study is comparable to that reported by Alebel (9.0%)^22^, Noubiap (8.0%)^23^ et al. in Africa. For smoking, our estimate is slightly higher than the prevalence among people with TB in Bangladesh and Pakistan (23%) which are TB high-burdened countries^69^. This is likely because their study focused on daily and current smoking only.

Our findings for the prevalence of hypertension and obesity are in agreement with the global estimates for the prevalence of hypertension (0.7–38.3%)^70^ and obesity (5.9%)^20^ in active TB. However, these estimates are lower than what is reported in the general population for hypertension (30.0–42.0%) and obesity (21.0%) in Africa^71,72^. A low prevalence of obesity in active TB should be expected since undernourishment accounts for the most global TB cases^5^ while obesity is protective against active TB^20^. With regards to hypertension, it is likely that HIV, age and obesity status influence the blood pressure dynamics in TB. HIV has been associated with 25% lower risk for hypertension^73^. In our meta-analysis for hypertension, HIV co-infection was reported among > 30% of participants in eight of the twelve studies for which HIV status data were reported, although estimates of hypertension by HIV status were not reported in most of the studies. Additionally, TB is predominantly a disease of young individuals while hypertension is more prevalent in older individuals in Africa^5,74^. Despite the relatively low prevalence of hypertension in active TB in Africa, screening is warranted due to the low levels of hypertension awareness, treatment initiation and blood pressure (Bp) control in Africa^75^. The low rate of hypertension treatment rates could explain why hypertension was most prevalent in East Africa—where the rate of hypertension treatment is lowest in Africa^75^.

Estimating the prevalence of alcohol use in Africa is difficult. This is because the types of alcoholic drinks are varied (often home brewed) and few studies use high-quality quantification of alcohol use^76^. Further, over 60% of “current drinkers” in Africa are heavy episodic drinkers (defined as consumption of ≥ 60 g of pure alcohol on at least one occasion at least once per month)^77^. Therefore, our estimate of the prevalence of any current alcohol use may not be very reliable. However, the estimate for hazardous alcohol use is likely to be more reliable. All studies (except five) included in the analysis for hazardous alcohol use used validated tools or quantified alcohol use by amount and/or frequency. Our estimate of hazardous alcohol use is similar to the prevalence of alcohol use disorder among people with TB in Africa (24.0%) reported by Necho et al.^78^. Alcohol use increases the risk for TB infection^16^ and poor TB outcomes^76^. Therefore, the high prevalence of hazardous alcohol use suggests a need for integrating validated tools in screening for alcohol use disorders among people at risk of TB and people with TB.

Our estimates should be interpreted in the context of some limitations. Firstly, there was significant heterogeneity and publication bias across most estimates. Therefore, the overall pooled estimates were over-estimated particularly due to publication bias. Our sensitivity analyses showed lower prevalence among studies within the funnel plots. Secondly, the prevalence for smoking may have been overestimated since any history of smoking (other than current smoking only) was considered in the pooled prevalence. However, the CVR posed by smoking is higher in former smokers than “never smokers”^79,80^. Therefore, assessing for any history of smoking as a CVR factor is justified. Moreover, half of the studies included in our analysis reported current smoking. Thirdly, transient hyperglycaemia can confound the estimates for DM. In our analysis, two studies demonstrated a decline in the prevalence of DM when measurements are taken at baseline and months into TB treatment^47,81^. Other two studies that assessed the prevalence at least a month into TB treatment report a prevalence of 2–3%^82,83^. Therefore, transient hyperglycaemia could spuriously increase the prevalence of DM. Nonetheless, hyperglycaemia does not resolve at follow-up in 50% of people with TB and hyperglycaemia at baseline^84^. More studies are needed to determine whether transient hyperglycaemia in TB heralds new onset DM or predicts future risk for DM. Regarding hypertension, none of the studies purposely set out to determine the prevalence of hypertension. Further, some studies based the diagnosis on single blood pressure measurements^59,85^, and the criteria were unknown in some other studies^54,57,62^. There is therefore a risk of selection and misclassification bias in the estimate for the prevalence of hypertension. Nonetheless the range of the prevalence of hypertension in our analysis is similar to that reported by Seegert et al.^70^ in a global systematic review. Importantly, CVR factors in Africa may be different to those in high-income countries. As such, the synergistic effect of aging, stress, illiteracy, poor health systems and poverty on CVD in Africa needs to be further evaluated among people with active TB^86^.

Notwithstanding the limitations, this analysis provides the first comprehensive prevalence of most traditional CVR factors among people with active TB in Africa. We further explored differences by TB drug resistance status and regions of Africa in the sub-analyses. Moreover, our study provides an updated estimate for the prevalence of DM in TB in Africa.

In conclusion, the prevalence of smoking, hazardous alcohol use and DM was high among people with TB in Africa. The findings suggest a need for screening for these CVR factors in this population. Although the prevalence of hypertension was low relative to regional estimates, screening is warranted because of the low awareness levels previously reported in the region. Prospective studies are needed to determine the role of CVR scoring among people with TB to prevent future CVD. More studies evaluating the burden of lipid abnormalities and physical inactivity among people with TB in Africa are needed as well.

## Supplementary Information

Supplementary Information.

## Data Availability

All data generated or analysed during this study are included in this article and its supplementary information files.
