# Supplementary material for: Prevalence of cardiovascular risk factors in active tuberculosis in Africa: a systematic review and meta-analysis
Source: Sci Rep. 2022 Sep 29;12:16354. doi: 10.1038/s41598-022-20833-0 (PMC9523035; doi:10.1038/s41598-022-20833-0)
Supplement: Supplementary file 1 — Supplementary Information. [file 41598_2022_20833_MOESM1_ESM.docx]

**SUPPLEMENTARY MATERIAL**

**Prevalence of cardiovascular risk factors in active tuberculosis in Africa: a systematic review and meta-analysis**

Joseph Baruch Baluku, MMed, MSc^1,2,†^, Olum Ronald, MBChB^3^, Peace Bagasha, MMed^3^, Emmy Okello, MMed, PhD^4^, Felix Bongomin, MSc^3,5^.

^1^Division of Pulmonology, Kiruddu National Referral Hospital, Kampala, Uganda
^2^Makerere University Lung Institute, Kampala, Uganda
^3^Department of Internal Medicine, School of Medicine, Makerere University College of Health Sciences, Kampala, Uganda
^4^Uganda Heart Institute, Kampala, Uganda
^5^Department of Medical Microbiology and Immunology, Faculty of Medicine, Gulu University, Gulu, Uganda

^†^Corresponding author
Dr. Joseph Baruch Baluku
PO Box 26343 Kampala, Uganda
bbjoe18@gmail.com
+256706327972

Table of Contents

[Characteristics of studies included in the analysis per cardiovascular risk factor 3](#_Toc110812001)

[Table 1: Characteristics of studies reporting the prevalence of smoking among people with active TB in Africa. 3](#_Toc110812002)

[Table 2: Characteristics of studies reporting the prevalence of alcohol use among people with active TB in Africa 6](#_Toc110812003)

[Table 3: Characteristics of studies reporting the prevalence of diabetes mellitus among people with active TB in Africa 10](#_Toc110812004)

[Table 4: Characteristics of studies reporting the prevalence of hypertension among people with active TB in Africa 13](#_Toc110812005)

[Table 5: Characteristics of studies reporting the prevalence of obesity among people with active TB in Africa 15](#_Toc110812006)

[Forrest plots for sub-analysis of studies falling within the funnel plots 16](#_Toc110812007)

[Figure 1: Sub-analysis for prevalence of smoking in people with TB among studies within the funnel plot 16](file:///C:\Users\joseph.baluku\Desktop\moi\Langh\CVD%20TB%20R1\Supplementary%20material%20R1.docx#_Toc110812008)

[Figure 3: Sub-analysis for prevalence of hazardous alcohol use in people with TB among studies within the funnel plot 17](file:///C:\Users\joseph.baluku\Desktop\moi\Langh\CVD%20TB%20R1\Supplementary%20material%20R1.docx#_Toc110812009)

[Figure 2: Sub-analysis for prevalence of current alcohol use among TB patients 17](file:///C:\Users\joseph.baluku\Desktop\moi\Langh\CVD%20TB%20R1\Supplementary%20material%20R1.docx#_Toc110812010)

[Figure 4: Sub-analysis for prevalence of diabetes mellitus in people with TB among studies within the funnel plot 18](file:///C:\Users\joseph.baluku\Desktop\moi\Langh\CVD%20TB%20R1\Supplementary%20material%20R1.docx#_Toc110812011)

[Figure 5: Sub-analysis for prevalence of obesity in people with TB among studies within the funnel plot 18](file:///C:\Users\joseph.baluku\Desktop\moi\Langh\CVD%20TB%20R1\Supplementary%20material%20R1.docx#_Toc110812012)

[Figure 6: Sub-analysis for prevalence of hypertension in people with TB among studies within the funnel plot 19](file:///C:\Users\joseph.baluku\Desktop\moi\Langh\CVD%20TB%20R1\Supplementary%20material%20R1.docx#_Toc110812013)

[Figure 7: PRISMA flow diagram 20](file:///C:\Users\joseph.baluku\Desktop\moi\Langh\CVD%20TB%20R1\Supplementary%20material%20R1.docx#_Toc110812014)

[Figure 8: Funnel plots for assessing publication bias for prevalence of cardiovascular risk factors in people with active TB in Africa 21](file:///C:\Users\joseph.baluku\Desktop\moi\Langh\CVD%20TB%20R1\Supplementary%20material%20R1.docx#_Toc110812015)

[Table 6: Risk of bias scores using Hoy and colleagues’ tool [111] 22](#_Toc110812016)

[Table 7: Studies excluded after full text review 27](#_Toc110812017)

[Table 8: Meta Regression analyses for prevalence of cardiovascular risk factors among people with active TB in Africa 28](#_Toc110812018)

[Table 9: Prevalence of cardiovascular risk factors among people with active TB in Africa after non-parametric trim and fill. 31](#_Toc110812019)

[Figure 9: Contour-enhanced funnel plots after trim and fill for assessing publication bias for prevalence of cardiovascular risk factors in people with active TB in Africas 32](file:///C:\Users\joseph.baluku\Desktop\moi\Langh\CVD%20TB%20R1\Supplementary%20material%20R1.docx#_Toc110812020)

[Table 10: Search Strategy 33](#_Toc110812021)

[REFERENCES 37](#_Toc110812022)

# Characteristics of studies included in the analysis per cardiovascular risk factor

## Table 1: Characteristics of studies reporting the prevalence of smoking among people with active TB in Africa.

| **Authors** | **Country** | **Region** | **Year of publication** | **Study design** | **Number of participants** | **Drug sensitivity status**  **(drug sensitive or resistant TB)** | **Criteria for smoking** |
| --- | --- | --- | --- | --- | --- | --- | --- |
| Sitas et al. (2004) [1] | South Africa | Southern Africa | 2004 | Case-control | 414 | Unknown | Use in the las 5 years |
| Sonnenberg et al. (2000) [2] | South Africa | Southern Africa | 2000 | Case-control | 418 | Unknown | Current use |
| Iradukunda et al. (2021) [3] | Burundi | East Africa | 2021 | Case-control | 180 | Mixed | Unknown |
| Wekunda et al. (2021) [4] | Kenya | East Africa | 2021 | Observational cross sectional | 291 | Unknown | Unknown |
| Petersen, et al. (2021) [5] | South Africa | Southern Africa | 2021 | Randomised controlled trial | 716 | Unknown | Ever smoked |
| Nyasulu et al. (2015) [6] | South Africa | Southern Africa | 2015 | Case-control | 100 | Unknown | Current use |
| Appiah, et al. (2021) [7] | Ghana | West Africa | 2021 | Observational cross sectional | 146 | Unknown | Ever smoked |
| Hill et al. (2006) [8] | Gambia | west Africa | 2006 | Case-control | 100 | Unknown | current use (within 6 months) |
| Boillat-Blanco et al.(2016) [9] | Tanzania | East Africa | 2016 | Case-control | 530 | Unknown | Ever smoked |
| Ali et al. (2019) [10] | Sudan | Northern Africa | 2019 | Case-control | 183 | Mixed | Unknown |
| Murrison et al. (2016) [11] | South Africa | Southern Africa | 2016 | Case-control | 133 | Mixed | Current use |
| Racil et al. (2012) [12] | Tunisia | Northern Africa | 2012 | Case-control | 185 | Unknown | ≥20 pack years |
| Deetlefts et al. (2012) [13] | South Africa | Southern Africa | 2012 | Cohort | 72 | Unknown | ever smoked |
| Kubjane et al. (2020) [14] | South Africa | Southern Africa | 2020 | Cohort | 400 | Mixed | current use |
| Magee et al. (2017) [15] | South Africa | Southern Africa | 2017 | Cohort | 91 | Drug resistant | current use |
| Otwombe et al. (2013) [16] | South Africa | Southern Africa | 2013 | Cohort | 891 | Unknown | current use |
| Iweama et al. (2021) [17] | Nigeria | West Africa | 2021 | Observational cross sectional | 390 | Mixed | Ever smoked |
| Wotale et al. (2021) [18] | Ethiopia | East Africa | 2021 | Observational cross sectional | 367 | Drug resistant | Unknown |
| Tulu, et al. (2021) [19] | Ethiopia | East Africa | 2021 | Observational cross sectional | 269 | Unknown | Current use |
| Assal, et al. (2021)[20] | Egypt | Northern Africa | 2021 | Cohort | 51 | Unknown | Unknown |
| Zetola et al (2021) [21] | Botswana | Southern Africa | 2021 | Observational cross sectional | 3736 | Mixed | Current smoking |
| Mitrani, et al (2021) [22] | South Africa | Southern Africa | 2021 | Observational cross sectional | 194 | Drug resistant | Unknown |
| Seegert et al. (2021) [23] | Guinea-Bissau | West Africa | 2021 | Cohort | 1541 | Unknown | current use |
| Sidamo et al. (2021) [24] | Ethiopia | East Africa | 2021 | Observational cross sectional | 80 | Drug resistant | Unknowns |
| Isralls et al. (2021) [25] | South Africa | Southern Africa | 2021 | Cohort | 420 | Drug resistant | Unknown |
| Balkissou et al. (2015) [26] | Cameroon | West Africa | 2015 | Cohort | 193 | Unknown | 20 pack years or 1 cigarette per day for 1 year |
| Whitehouse et al. (2019) [27] | South Africa | Southern Africa | 2019 | Cross sectional nested in randomised controlled trial (RCT) | 891 | Drug resistant | >100 lifetime cigarettes |
| Yorke et al. (2018) [28] | Ghana | West Africa | 2018 | Observational cross sectional | 146 | Unknown | unknown |
| Gebrehiwet et al. (2019) [29] | Ethiopia | East Africa | 2019 | Observational cross sectional | 94 | Mixed | unknown |
| Ayeni, et al. (2021) [30] | Nigeria | West Africa | 2021 | Observational Cross sectional | 671 | Unknown | Unknown |
| Farghaly et al. (2021)[31] | Egypt | Northern Africa | 2021 | Observational cross sectional | 88 | Mixed | Ever smoked |
| Baik et al. (2020) [32] | South Africa | Southern Africa | 2020 | Cross sectional nested in RCT | 699 | Unknown | Current use |
|  | Uganda | East Africa | 2020 | Cross sectional nested in RCT | 108 | Unknown | Current use |
| Asante-Poku et al. (2019) [33] | Ghana | West Africa | 2019 | Observational cross sectional | 2990 | Unknown | ever smoked |
| Mabula et al. (2021) [34] | Tanzania | East Africa | 2021 | Observational Cross sectional | 153 | Unknown | Ever smoked |
| Tiamiyu et al. (2020) [35] | Nigeria | west Africa | 2020 | Observational cross sectional | 150 | Unknown | >100 lifetime cigarettes |
| Van't Hoog et al. (2013) [36] | Kenya | East Africa | 2013 | Observational cross sectional | 272 | Unknown | ever smoked |
| Musuenge et al. (2020) [37] | Burkina Faso | west Africa | 2020 | Observational cross sectional | 302 | Unknown | current use |
| Kirenga et al. (2015) [38] | Uganda | East Africa | 2015 | Observational cross sectional | 365 | Mixed | >100 lifetime cigarettes |
| Adegbite et al. (2020) [39] | Gabon | West-central Africa | 2020 | Observational cross sectional | 295 | Unknown | unknown |
| Lam et al. (2013) [40] | South Africa | Southern Africa | 2013 | Observational cross sectional | 707 | Unknown | <2 months |
| Ugarte-Gil et al. (2020) [41] | South Africa | Southern Africa | 2020 | Observational cross sectional | 331 | Unknown | current use |
| Faurholt-Jepsen et al. (2012) [42] | Tanzania | East Africa | 2012 | Observational cross sectional | 1025 | Unknown | current use |
| Kibirige et al. (2013) [43] | Uganda | east Africa | 2013 | Observational cross sectional | 260 | Unknown | ever smoked |
| Pizzol et al. (2017) [44] | Mozambique | Southern Africa | 2017 | Observational cross sectional | 294 | Unknown | 1-20 sticks/day |
| Mollel et al. (2017) [45] | Tanzania | East Africa | 2017 | Observational cross sectional | 147 | Drug resistant | unknown |
| Kassa et al. (2021) [46] | Ethiopia | East Africa | 2021 | Cohort | 580 | Drug resistant | Ever smoked |
| Louwagie et al. (2013) [47] | South Africa | Southern Africa | 2013 | Observational cross sectional | 1924 | Unknown | current use |
| Mulisa et al. (2015) [48] | Ethiopa | East Africa | 2015 | Observational cross sectional | 263 | Mixed | unknown |
| Segafredo et al. (2019) [49] | Angola | Southern Africa | 2019 | Observational cross sectional | 7132 | Unknown | ever smoked |
| Tola et al. (2015) [50] | Ethiopa | East Africa | 2015 | Observational cross sectional | 330 | Mixed | West Australian Government Smoking Assessment Checklist |
| Adetifa et al. (2017) [51] | Gambia | west Africa | 2017 | Observational cross sectional | 248 | Unknown | unknown |
| Shangase et al. (2018) [52] | South Africa | Southern Africa | 2018 | Observational cross sectional | 211 | Drug resistant | current use |
| Oni et al. (2017) [53] | South Africa | Southern Africa | 2017 | Observational cross sectional | 400 | Unknown | current use |
| Nagu et al. (2017) [54] | Tanzania | East Africa | 2017 | Observational cross sectional | 252 | Unknown | current use |
| Haraldsdottir et al. (2015) [55] | Guinea-Bissau | west Africa | 2015 | Observational cross sectional | 110 | Unknown | current use |
| Kufa et al. (2016) [56] | South Africa | Southern Africa | 2016 | Observational cross sectional | 15 | Unknown | ever smoked |
| Dalton et al. (2012) [57] | South Africa | Southern Africa | 2012 | Observational cross sectional | 292 | Drug resistant | current use |
| Sattar et al. (2014) [58] | South Africa and Zambia | Southern Africa | 2014 | Observational cross sectional | 894 | Unknown | current use |
| Molalign et al. (2015) [59] | Ethiopia | East Africa | 2015 | Observational cross sectional | 342 | Drug resistant | unknown |
| Fwoloshi et al. (2018) [60] | Zambia | Southern Africa | 2018 | Observational cross sectional | 119 | Unknown | unknown |
| Diande et al. (2019) [61] | Burkina Faso | west Africa | 2019 | Observational cross sectional | 1140 | Mixed | unknown |
| Ade et al. (2015) [62] | Benin | west Africa | 2015 | Observational cross sectional | 159 | Mixed | Unknown |
| Mwiru et al. (2017) [63] | Tanzania | East Africa | 2017 | Observational cross sectional | 518 | Unknown | ever smoked |
| Naidoo et al. (2013) [64] | South Africa | Southern Africa | 2013 | Observational cross sectional | 3050 | Unknown | current use |
| Munseri et al. (2019) [65] | Tanzania | East Africa | 2019 | Observational cross sectional | 660 | Unknown | ever smoked |
| Stracker et al. (2019) [66] | south Africa | Southern Africa | 2019 | Observational cross sectional nested in clustered randomised trial | 318 | Unknown | ever smoked |
| Ekeke et al. (2017) [67] | Nigeria | west Africa | 2017 | Observational cross sectional | 2094 | Unknown | current use |
| Berkowitz et al. (2018) [68] | South Africa | Southern Africa | 2018 | Observational cross sectional | 13 | Unknown | ever smoked |
| Tekie-Desta et al. (2018) [69] | Liberia | west Africa | 2018 | Observational cross sectional | 139 | Unknown | current use (within 6 months) |
| Peltzer et al. (2013) [70] | South Africa | Southern Africa | 2013 | Observational cross sectional | 4900 | Unknown | current use |
| Ngosa et al. (2016) [71] | Zambia | Southern Africa | 2016 | Observational cross sectional | 34 | Unknown | current use |
| Gunasekera et al. (2020) [72] | South Africa | Southern Africa | 2020 | Observational cross sectional | 660 | Unknown | current use |
| Wessels et al. (2019) [73] | South Africa | Southern Africa | 2019 | Observational cross sectional | 100 | Unknown | current use |
| Mburu et al. (2018) [74] | Kenya | East Africa | 2018 | Observational cross sectional | 347 | Unknown | ever used |
| Kombila et al. (2017) [75] | Senegal | west Africa | 2017 | Observational cross sectional | 165 | Unknown | current use |
| Brunet et al. (2011) [76] | South Africa | Southern Africa | 2011 | Observational cross sectional nested in cohort | 286 | Unknown | current use |
| Azeez et al. (2018) [77] | South Africa | Southern Africa | 2018 | Retrospective cohort | 910 | Drug resistant | ever smoked |
| Watermeyer et al. (2018) [78] | South Africa | Southern Africa | 2018 | Retrospective cohort | 68 | Unknown | current use |
| Kootbodien et al. (2018) [79] | South Africa | Southern Africa | 2018 | Retrospective cohort | 187636 | Unknown | ever smoked |

## Table 2: Characteristics of studies reporting the prevalence of alcohol use among people with active TB in Africa

| **Authors** | **Country** | **Geographical region** | **Year of publication** | **Study design** | **Number of participants** | **Drug sensitivity status**  **(drug sensitive or resistant TB)** | **Criteria for alcohol use** |
| --- | --- | --- | --- | --- | --- | --- | --- |
| Ali et al. (2019) [10] | Sudan | Northern Africa | 2019 | Case-control | 183 | Mixed | Chronic drinker |
| Soboka et al. (2021) [80] | Ethiopia | East Africa | 2021 | Cohort | 268 | Drug sensitive | AUDIT ≥8 |
| Dayyab, et al. (2021) [81] | Nigeria | West Africa | 2021 | Cohort | 39 | Drug resistant | Unknow |
| Mitrani, et al (2021) [22] | South Africa | Southern Africa | 2021 | Observational cross sectional | 194 | Drug resistant | “Misusing alcohol” |
| Boillat-Blanco et al. (2016) [9] | Tanzania | East Africa | 2016 | Case-control | 530 | Unknown | Alcohol misuse (≥3 drinks per day or ≥6 drinks per occasion) |
| Kassa et al. (2021) [46] | Ethiopia | East Africa | 2021 | Cohort | 578 | Drug resistant | Unknown |
| Appiah, et al. (2021) [7] | Ghana | West Africa | 2021 | Observational cross sectional | 146 | Unknown | Ever used |
| Wekunda et al. (2021) [4] | Kenya | East Africa | 2021 | Observational cross sectional | 291 | Unknown | Unknown |
| Iradukunda et al. (2021) [3] | Burundi | East Africa | 2021 | Case-control | 180 | Mixed | Unknown |
| Sidamo et al. (2021) [24] | Ethiopia | East Africa | 2021 | Observational cross sectional | 80 | Drug resistant | Unknown |
| Ayeni, et al. (2021) [30] | Nigeria | West Africa | 2021 | Observational Cross sectional | 671 | Unknown | Unknown |
| Wotale et al. (2021) [18] | Ethiopia | East Africa | 2021 | Observational cross sectional | 367 | Drug resistant | Unknown |
| Soboka et al. (2021) [82] | Ethiopia | East Africa | 2021 | Case-control | 268 | Unknown | AUDIT ≥8 |
| Tulu et al. (2021) [19] | Ethiopia | East Africa | 2021 | Observational cross sectional | 269 | Unknown | Regular use |
| Oumer et al. (2021) [83] | Ethiopia | East Africa | 2021 | Case-control | 450 | Mixed | Unknown |
| Murrison et al. (2016) [11] | South Africa | Southern Africa | 2016 | Case-control | 133 | Mixed | Heavy drinking: ≥ 15 drinks/week |
| Zetola et al. (2012) [84] | Botswana | Southern Africa | 2012 | Case-control | 228 | Mixed | Current use |
| Racil et al. (2012) [12] | Tunisia | Northern Africa | 2012 | Case-control | 185 | Unknown | Daily use |
| Hill et al. (2006) [8] | Gambia | West Africa | 2006 | Case-control | 100 | Unknown | Ever used |
| Sonnenberg (2000) [2] | South Africa | Southern Africa | 2000 | Case-control | 418 | Unknown | Current use (11.1% had daily use) |
| Peltzer et al. (2013) [85] | South Africa | Southern Africa | 2013 | Clustered randomised controlled trial (RCT) | 4880 | Unknown | AUDIT ≥8 for men and AUDIT of ≥7 for women |
| Kubjane et al. (2020) [14] | South Africa | Southern Africa | 2020 | Cohort | 412 | Mixed | Binge drinking |
| Magee et al. (2017) [15] | South Africa | Southern Africa | 2017 | Cohort | 91 | Drug resistant | Current use |
| Balkissou et al. (2015) [26] | Cameroon | West Africa | 2015 | Cohort | 193 | Unknown | Chronic alcoholism |
| Otwombe et al. (2013) [16] | South Africa | Southern Africa | 2013 | Cohort | 891 | Unknown | Current use |
| Gebrehiwet et al. (2019) [29] | Ethiopa | East Africa | 2019 | Observational cross sectional | 94 | Mixed | Unknown |
| Yorke et al. (2018) [28] | Ghana | West Africa | 2018 | Observational cross sectional | 146 | Unknown | Unknown |
| Churchyard et al. (2010) [86] | South Africa | Southern Africa | 2010 | Cross sectional nested in RCT | 319 | Unknown | 1- 28 units/ week |
| Musuenge et al. (2020) [37] | Burkina Faso | West Africa | 2020 | Observational cross sectional | 302 | Unknown | Current use |
| Gunasekera et al. (2020) [72] | South Africa | Southern Africa | 2020 | Observational cross sectional | 660 | Unknown | Daily use |
| Mabula et al. (2021) [34] | Tanzania | East Africa | 2021 | Observational Cross sectional | 153 | Unknown | Ever used |
| Asante-Poku et al. (2019) [33] | Ghana | West Africa | 2019 | Observational cross sectional | 2990 | Unknown | Current use |
| Seegert et al. (2021) [23] | Guinea-Bissau | West Africa | 2021 | Cohort | 1400 | Unknown | Daily use |
| Segafredo et al. (2019) [49] | Angola | Southern Africa | 2019 | Observational cross sectional | 7150 | Unknown | Unknown |
| Diande et al. (2019) [61] | Burkina Faso | West Africa | 2019 | Observational cross sectional | 1140 | Mixed | Unknown |
| Munseri et al. (2019) [65] | Tanzania | East Africa | 2019 | Observational cross sectional | 660 | Unknown | Current use |
| Wessels et al. (2019) [73] | South Africa | Southern Africa | 2019 | Observational cross sectional | 100 | Unknown | >3 days per week |
| Berkowitz et al. (2018) [68] | South Africa | Southern Africa | 2018 | Observational cross sectional | 13 | Unknown | Current use |
| Tekie-Desta et al. (2018) [69] | Liberia | West Africa | 2018 | Observational cross sectional | 139 | Unknown | Current use (within 6 months) |
| Mburu et al. (2018) [74] | Kenya | East Africa | 2018 | Observational cross sectional | 347 | Unknown | Current use |
| Bhana et al. (2017) [87] | South Africa | Southern Africa | 2017 | Observational cross sectional | 73 | Unknown | AUDIT ≥8 |
| Wanyonyi et al. (2017) [88] | Kenya | East Africa | 2017 | Observational cross sectional | 252 | Unknown | >3 days per week |
| Pizzol (2017) [44] | Mozambique | Southern Africa | 2017 | Observational cross sectional | 294 | Unknown | Current use |
| Hayes-Larson et al. (2017) [89] | Lesotho | Southern Africa | 2017 | Observational cross sectional | 364 | Unknown | AUDIT ≥8 |
| Mollel et al. (2017) [45] | Tanzania | East Africa | 2017 | Observational cross sectional | 147 | Drug resistant | Unknown |
| Nagu et al. (2017) [54] | Tanzania | East Africa | 2017 | Observational cross sectional | 252 | Unknown | Current use |
| Mwiru et al. (2017) [63] | Tanzania | East Africa | 2017 | Observational cross sectional | 518 | Unknown | Heavy drinking: > 1 drink per day for women and >2 drinks per day for men |
| Kombila et al. (2017) [75] | Senegal | West Africa | 2017 | Observational cross sectional | 165 | Unknown | Unknown |
| Kufa et al. (2016) [56] | South Africa | Southern Africa | 2016 | Observational cross sectional | 15 | Unknown | Current use |
| Iweama et al. (2021) [17] | Nigeria | West Africa | 2021 | Observational cross sectional | 390 | Mixed | Current use |
| Zetola et a. (2021) [21] | Botswana | Southern Africa | 2021 | Observational cross sectional | 3736 | Mixed | ≥5 drinks/session or drinking on >5 days/month |
| Kirenga (2015) [38] | Uganda | East Africa | 2015 | Observational cross sectional | 365 | Mixed | >3 days per week |
| Mulisa et al. (2015) [48] | Ethiopa | East Africa | 2015 | Observational cross sectional | 262 | Mixed | Chronic drinker |
| Tola et al. (2015) [50] | Ethiopa | East Africa | 2015 | Observational cross sectional | 330 | Mixed | AUDIT ≥8 |
| Haraldsdottir et al. (2015) [55] | Guinea-Bissau | West Africa | 2015 | Observational cross sectional | 110 | Unknown | Daily use |
| Van't-Hoog et al. (2013) [36] | Kenya | East Africa | 2013 | Observational cross sectional | 271 | Unknown | Current use |
| Lam et al. (2013) [40] | South Africa | Southern Africa | 2013 | Observational cross sectional | 707 | Unknown | Daily use |
| Louwagie et al. (2013) [47] | South Africa | Southern Africa | 2013 | Observational cross sectional | 1895 | Unknown | CAGE score of ≥2 |
| O'Connell et al. (2013) [90] | Zambia | Southern Africa | 2013 | Observational cross sectional | 355 | Unknown | Alcohol dependence using Mini International Neuropsychiatric Interview |
| Naidoo et al. (2013) [64] | South Africa | Southern Africa | 2013 | Observational cross sectional | 3107 | Unknown | AUDIT ≥8 |
| Peltzer et al. (2013) [70] | South Africa | Southern Africa | 2013 | Observational cross sectional | 4900 | Unknown | AUDIT ≥8 |
| Matseke et al. (2012) [91] | South Africa | Southern Africa | 2012 | Observational cross sectional | 4808 | Unknown | AUDIT ≥8 |
| Dalton et al. (2012) [57] | South Africa | Southern Africa | 2012 | Observational cross sectional | 293 | Drug resistant | Alcohol abuse |
| Muture et al. (2011) [92] | Kenya | East Africa | 2011 | Observational cross sectional | 274 | Unknown | Unknown |
| Talbot et al. (2002) [93] | Botswana | Southern Africa | 2002 | Observational cross sectional | 135 | Unknown | >3 drinks/day |
| Conradie et al. (2013) [94] | South Africa | Southern Africa | 2013 | Cohort | 246 | Drug resistant | >3 times a week |
| Azeez et al. (2018) [77] | South Africa | Southern Africa | 2018 | Cohort | 910 | Drug resistant | Current use |
|  |  |  |  |  |  |  |  |

## Table 3: Characteristics of studies reporting the prevalence of diabetes mellitus among people with active TB in Africa

| **Authors** | **Country** | **Geographical region** | **Year of publication** | **Study design** | **Number of participants** | **Drug sensitivity status**  **(drug sensitive or resistant TB)** | **Criteria for diagnosis** |
| --- | --- | --- | --- | --- | --- | --- | --- |
| Assal, et al. (2021)[20] | Egypt | Northern Africa | 2021 | Cohort | 51 | Unknown | Self-report (known history of diabetes mellitus (DM)) |
| Mitrani, et al (2021) [22] | South Africa | Southern Africa | 2021 | Observational cross sectional | 194 | Drug resistant | Unknown |
| Ayeni, et al. (2021) [30] | Nigeria | West Africa | 2021 | Observational Cross sectional | 671 | Unknown | FBS ≥126mg/dl or HbA1c≥6.5% after RBS ≥200 mg/dl |
| Isralls et al. (2021) [25] | South Africa | Southern Africa | 2021 | Cohort | 420 | Drug resistant | Unknown |
| Iradukunda et al. (2021) [3] | Burundi | East Africa | 2021 | Case-control | 180 | Mixed | Unknown |
| Kebede, et al. (2021) [95] | Ethiopia | East Africa | 2021 | Observational cross sectional | 465 | Unknown | Unknown |
| Wotale et al. (2021) [18] | Ethiopia | East Africa | 2021 | Cohort | 367 | Drug resistant | Unknown |
| Kirubi, et ak. (2021) [96] | Kenya | East Africa | 2021 | Observational cross sectional | 1048 | Drug sensitive | Unknown |
| Tulu, et al (2021)[19] | Ethiopia | East Africa | 2021 | Observational cross sectional | 269 | Unknown | FBS ≥126mg/dl |
| Pillay, et al. (2021) [97] | South Africa | Southern Africa | 2021 | Cohort | 547 | Unknown | Unknown |
| Erias, et al. (2021) [98] | Uganda | East Africa | 2021 | Observational cross sectional | 187 | Unknown | HbA1c≥6.5% |
| Mabula, et al. (2021) [34] | Tanzania | East Africa | 2021 | Observational cross sectional | 153 | Unknown | FBS ≥126mg/dl after RBS ≥110 mg/dl |
| Musuenge et al. (2020) [37] | Burkina Faso | West Africa | 2020 | Observational cross sectional | 302 | Unknown | Self-report |
| Ugarte-Gil et al. (2020) [41] | South Africa | Southern Africa | 2020 | Observational cross sectional | 259 | Unknown | HbA1c ≥6.5% and known DM |
| Baik et al. (2020) [32] | South Africa | Southern Africa | 2020 | Cross sectional nested in RCT | 687 | Unknown | Self-report (known history of DM) |
|  | Uganda | East Africa | 2020 | Cross sectional nested in RCT | 104 | Unknown | Self-report (known history of DM) |
| Kubjane et al. (2020) [14] | South Africa | Southern Africa | 2020 | Cohort | 412 | Mixed | FBS ≥126 mg/dl or Hba1c ≥6.5% or known DM |
| Asante-Poku et al. (2019) [33] | Ghana | West Africa | 2019 | Observational cross sectional | 2990 | Unknown | HbA1c ≥6.5% |
| Whitehouse et al. (2019) [27] | South Africa | Southern Africa | 2019 | Cross sectional nested in RCT | 862 | Drug resistant | HbA1c ≥6.5% or DM medication or history of DM |
| Segafredo et al. (2019) [49] | Angola | Southern Africa | 2019 | Observational cross sectional | 7205 | Unknown | FBS ≥126 mg/dl or RBS ≥200 mg/dl or previous history DM |
| Ncube et al. (2019) [99] | Zimbabwe | Southern Africa | 2019 | Observational cross sectional | 427 | Unknown | FBS ≥126 mg/dl after RBS ≥110 mg/dl |
| Munseri et al. (2019) [65] | Tanzania | East Africa | 2019 | Observational cross sectional | 660 | Unknown | FBS ≥126 mg/dl or 2h PPG ≥200 mg/dl (after OGTT) or known DM |
| Ali et al. (2019) [10] | Sudan | Northern Africa | 2019 | Case-control | 183 | Mixed | Self-report (known history of DM) |
| Nsonga (2019) [100] | Uganda | East Africa | 2019 | Observational cross sectional | 4016 | Unknown | FBS ≥126mg/dl after RBS ≥110 mg/dl |
| Azeez et al. (2018) [77] | South Africa | Southern Africa | 2018 | Retrospective cohort | 910 | Drug resistant | Unknown |
| Yorke et al. (2018) [28] | Ghana | West Africa | 2018 | Cross sectional nested in RCT | 146 | Unknown | OGTT (PG ≥200 mg/dl) |
| Fwoloshi et al. (2018) [60] | Zambia | Southern Africa | 2018 | Observational cross sectional | 127 | Unknown | FBS ≥126mg/dl or known DM |
| Peltzer et al. (2018) [101] | South Africa | Southern Africa | 2018 | Observational cross sectional | 4207 | Unknown | Self-reports (known DM) |
| Mburu et al. (2018) [74] | Kenya | East Africa | 2018 | Observational cross sectional | 347 | Unknown | HbA1C >6.0% |
| Pizzol et al. (2017) [44] | Mozambique | Southern Africa | 2017 | Observational cross sectional | 299 | Unknown | FBS ≥126 mg/dl |
| McEbula et al. (2017) [102] | South Africa | Southern Africa | 2017 | Observational cross sectional | 325 | Unknown | OGTT (2h PPG ≥200 mg/dl) and known DM |
| Oni et al. (2017) [53] | South Africa | Southern Africa | 2017 | Observational cross sectional | 414 | Unknown | FBS ≥126 mg/dl, OGTT (2h PPG ≥200 mg/dl) or Hba1C ≥6.5% |
| Nagu et al. (2017) [54] | Tanzania | East Africa | 2017 | Observational cross sectional | 253 | Unknown | RBS ≥200 mg/dl or DM treatment |
| Owiti et al. (2017) [103] | Kenya | East Africa | 2017 | Observational cross sectional | 454 | Unknown | HbA1C ≥6.5% |
| Ekeke et al. (2017) [67] | Nigeria | West Africa | 2017 | Observational cross sectional | 2094 | Unknown | FBS ≥126mg/dl after RBS ≥110mg/dl |
| Lawson et al. (2017) [104] | Nigeria | West Africa | 2017 | Observational cross sectional | 113 | Unknown | HbA1C ≥6.5% or known DM |
| Boillat-Blanco et al. (2016) [9] | Tanzania | East Africa | 2016 | Case-control | 530 | Unknown | HbA1C ≥6.5% (chosen because prevalence by this test was more consistent even during TB treatment) |
| Kirenga et al. (2015) [38] | Uganda | East Africa | 2015 | Observational cross sectional | 130 | Mixed | FBS ≥126mg/dl |
| Umanah et al. (2015) [105] | South Africa | Southern Africa | 2015 | Retrospective cohort | 160 | Drug resistant | Unknown |
| Oni et al. (2015) [106] | South Africa | Southern Africa | 2015 | Observational cross sectional | 387 | Unknown | DM treatment |
| Haraldsdottir et al. (2015) [55] | Guinea-Bissau | West Africa | 2015 | Observational cross sectional | 107 | Unknown | FBS ≥126 mg/dl (if RBS was ≥216 mg/dl) |
| Molalign et al. (2015) [59] | Ethiopia | East Africa | 2015 | Observational cross sectional | 342 | Drug resistant | Unknown |
| Ade et al. (2015) [62] | Benin | West Africa | 2015 | Observational cross sectional | 159 | Mixed | FBS ≥126 mg/dl |
| Balkissou et al. (2015) [26] | Cameroon | West Africa | 2015 | Cohort | 193 | Unknown | Self-report (known history of DM) |
| Sattar et al. (2014) [58] | South Africa and Zambia | Southern Africa | 2014 | Observational cross sectional | 894 | Unknown | Self-report (known history of DM) |
| Kibirige et al. (2013) [43] | Uganda | East Africa | 2013 | Observational cross sectional | 260 | Unknown | RBS ≥200 mg/dl |
| Dalton et al. (2012) [57] | South Africa | Southern Africa | 2012 | Observational cross sectional | 293 | Drug resistant | Unknown |
| Faurholt-Jepsen et al. (2011) [107] | Tanzania | East Africa | 2011 | Observational cross sectional | 803 | Unknown | FBS > 108 mg/dl or a OGTT (2h PPG >198 mg/dl) |
| Hill et al. (2006) [8] | Gambia | West Africa | 2006 | Case-control | 100 | Unknown | Self-report (known history of DM) |
| Araia et al (2021) [108] | Eritrea | East Africa | 2021 | Observational cross sectional | 1134 | Unknown | FBS ≥126 mg/dl ( n = 51) and known DM ( n = 61) |

## Table 4: Characteristics of studies reporting the prevalence of hypertension among people with active TB in Africa

| **Authors** | **Year of publication** | **Country** | **Region** | **Study design** | **Number of participants** | **Drug sensitivity status**  **(drug sensitive or resistant TB)** | **Criteria for diagnosis** |
| --- | --- | --- | --- | --- | --- | --- | --- |
| Musuenge et al. (2020) [37] | 2020 | Burkina Faso | West Africa | Observational cross sectional | 302 | Unknown | Unknown |
| Dayyab, et al. (2021) [81] | 2021 | Nigeria | West Africa | Cohort | 39 | Drug resistant | Unknow |
| Seegert et al. (2021)[23] | 2021 | Guinea-Bissau | West Africa | Cohort | 1544 | Unknown | SBP >140 mmHg and/or DBP >90 mmHg |
| Wotale et al. (2021) [18] | 2021 | Ethiopia | East Africa | Cohort | 367 | Drug resistant | Unknown |
| Isralls et al. (2021) [25] | 2021 | South Africa | Southern Africa | Cohort | 420 | Drug resistant | Unknown |
| Ugarte-Gil et al. (2020) [41] | 2020 | South Africa | Southern Africa | Observational cross sectional | 331 | Unknown | Hypertension medication |
| Kubjane et al. (2020) [14] | 2020 | South Africa | Southern Africa | Cohort | 412 | Mixed | Systolic blood pressure (SBP) >140 mmHg and/or diastolic blood pressure (DBP) >90 mmHg or known hypertension |
| Mitrani, et al (2021) [22] | 2021 | South Africa | Southern Africa | Observational cross sectional | 194 | Drug resistant | Unknown |
| Whitehouse et al. (2019) [27] | 2019 | South Africa | Southern Africa | Cross sectional nested in RCT | 828 | Drug resistant | SBP >140 mmHg and/or DBP >90 mmHg or known hypertension history or hypertension medication. |
| Segafredo et al. (2019) [49] | 2019 | Angola | Southern Africa | Observational cross sectional | 6954 | Unknown | SBP >140 mmHg and/or DBP >90 mmHg |
| Peltzer et al. (2018) [101] | 2018 | South Africa | Southern Africa | Observational cross sectional | 4207 | Unknown | Known hypertension |
| Pizzol et al. (2017) [44] | 2017 | Mozambique | Southern Africa | Observational cross sectional | 294 | Unknown | Unknown |
| Oni et al. (2017) [53] | 2017 | South Africa | Southern Africa | Observational cross sectional | 414 | Unknown | SBP >140 mmHg and/or DBP >90 mmHg or known hypertension or hypertension medication. |
| Nagu et al. (2017) [54] | 2017 | Tanzania | East Africa | Observational cross sectional | 253 | Unknown | SBP >140 mmHg and/or DBP >90 mmHg or hypertension medication. |
| Owiti et al. (2017) [103] | 2017 | Kenya | East Africa | Observational cross sectional | 416 | Unknown | SBP >140 mmHg and/or DBP >90 mmHg or known hypertension or hypertension medication. |
| Boillat-Blanco et al. (2016) [9] | 2016 | Tanzania | East Africa | Case-control | 530 | Unknown | SBP >140 mmHg and/or DBP >90 mmHg or known hypertension or hypertension medication |
| Umanah et al. (2015) [105] | 2015 | South Africa | Southern Africa | Retrospective cohort | 160 | Drug resistant | Unknown |
| Oni et al. (2015) [106] | 2015 | South Africa | Southern Africa | Observational cross sectional | 387 | Unknown | Hypertension medication |
| Ade et al. (2015) [62] | 2015 | Benin | West Africa | Observational cross sectional | 159 | Mixed | Unknown |

## Table 5: Characteristics of studies reporting the prevalence of obesity among people with active TB in Africa

| **Study** | **Country** | **Geographical region** | **Year of publication** | **Study design** | **Number of participants** | **Drug Sensitivity** | **Criteria for diagnosis** |
| --- | --- | --- | --- | --- | --- | --- | --- |
| Araia et al. (2021) [108] | Eritrea | East Africa | 2021 | Observational cross sectional | 999 | Unknown | Body mass index (BMI*) ≥30 |
| Seegert et al. (2021)[23] | Guinea-Bissau | West Africa | 2021 | Cohort | 1520 | Unknown | BMI ≥30 |
| Appiah, et al. (2021) [7] | Ghana | West Africa | 2021 | Observational cross sectional | 146 | Unknown | BMI ≥30 |
| Erias, et al. (2021) [98] | Uganda | East Africa | 2021 | Observational cross sectional | 187 | Unknown | BMI ≥30 |
| Tiamiyu et al. (2020) [35] | Nigeria | West Africa | 2020 | Observational cross sectional | 150 | Unknown | BMI ≥30 |
| Ugarte-Gil et al. (2020) [41] | South Africa | Southern Africa | 2020 | Observational cross sectional | 331 | Unknown | BMI ≥30 |
| Kubjane et al. (2020) [14] | South Africa | Southern Africa | 2020 | Cohort | 398 | Mixed | BMI ≥30 |
| Whitehouse et al. (2019) [27] | South Africa | Southern Africa | 2019 | Cross sectional nested in RCT | 900 | Drug resistant | BMI ≥30 |
| Segafredo et al. (2019) [49] | Angola | Southern Africa | 2019 | Observational cross sectional | 6866 | Unknown | BMI ≥30 |
| Munseri et al. (2019) [65] | Tanzania | East Africa | 2019 | Observational cross sectional | 659 | Unknown | BMI ≥30 |
| Wessels et al. (2019) [73] | South Africa | Southern Africa | 2019 | Observational cross sectional | 100 | Unknown | BMI ≥30 |
| t'Veld et al. (2018) [109] | South Africa | Southern Africa | 2018 | Observational cross sectional | 119 | Unknown | BMI ≥30 |
| Yorke et al. (2018) [28] | Ghana | West Africa | 2018 | Observational cross sectional | 146 | Unknown | BMI ≥30 |
| Fwoloshi et al. (2018) [60] | Zambia | Southern Africa | 2018 | Observational cross sectional | 119 | Unknown | BMI ≥30 |
| Oni et al. (2017) [53] | South Africa | Southern Africa | 2017 | Observational cross sectional | 399 | Unknown | BMI ≥30 |
| Owiti et al. (2017) [103] | Kenya | East Africa | 2017 | Observational cross sectional | 445 | Unknown | BMI ≥30 |
| Bailey et al. (2016) [110] | Zambia | Southern Africa | 2016 | Observational cross sectional nested in a clustered RCT | 144 | Unknown | BMI ≥30 |
|  | South Africa | Southern Africa | 2016 | Observational cross sectional nested in a clustered RCT | 285 | Unknown | BMI ≥30 |

*in kilograms/meters^2^

# Forrest plots for sub-analysis of studies falling within the funnel plots


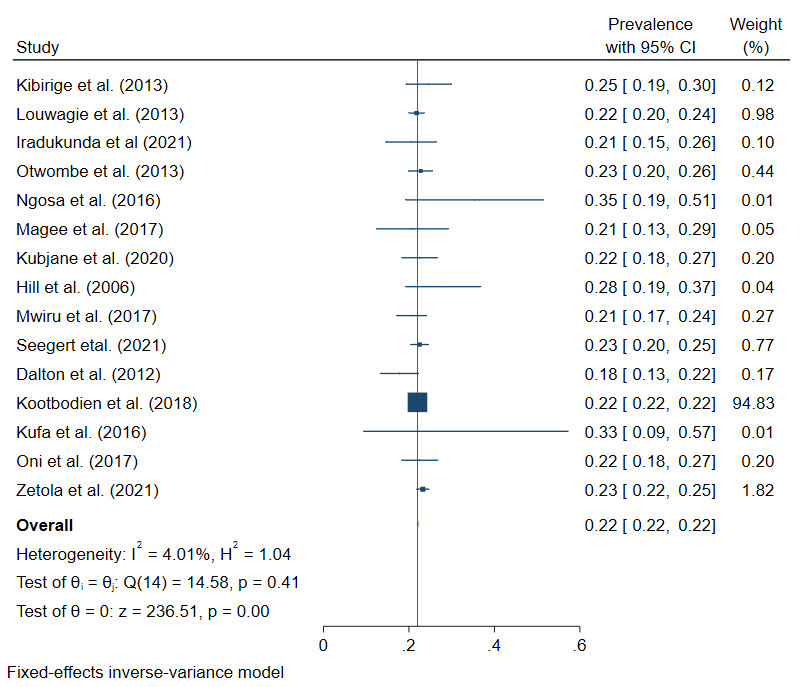


## Figure 1: Sub-analysis for prevalence of smoking in people with TB among studies within the funnel plot


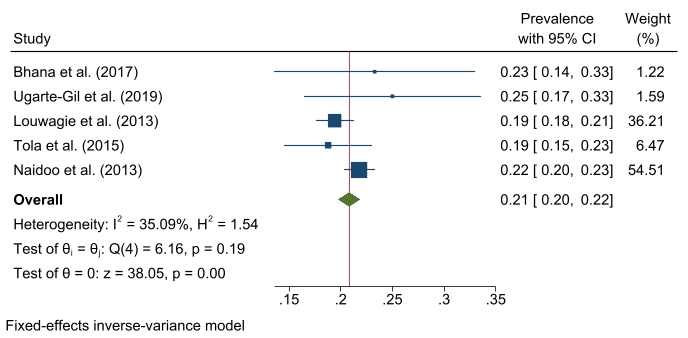

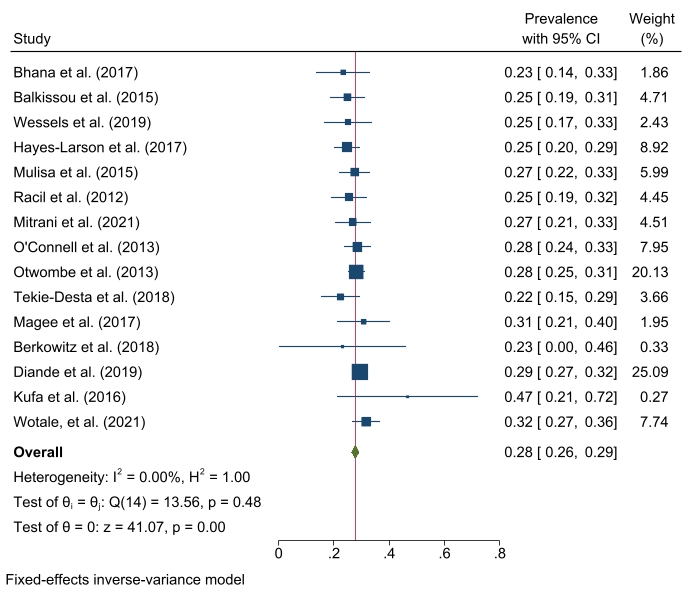


## Figure 3: Sub-analysis for prevalence of hazardous alcohol use in people with TB among studies within the funnel plot

## Figure 2: Sub-analysis for prevalence of current alcohol use among TB patients


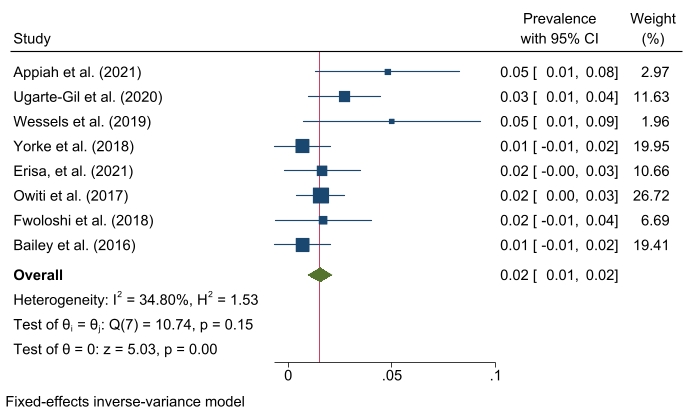


## Figure 4: Sub-analysis for prevalence of diabetes mellitus in people with TB among studies within the funnel plot


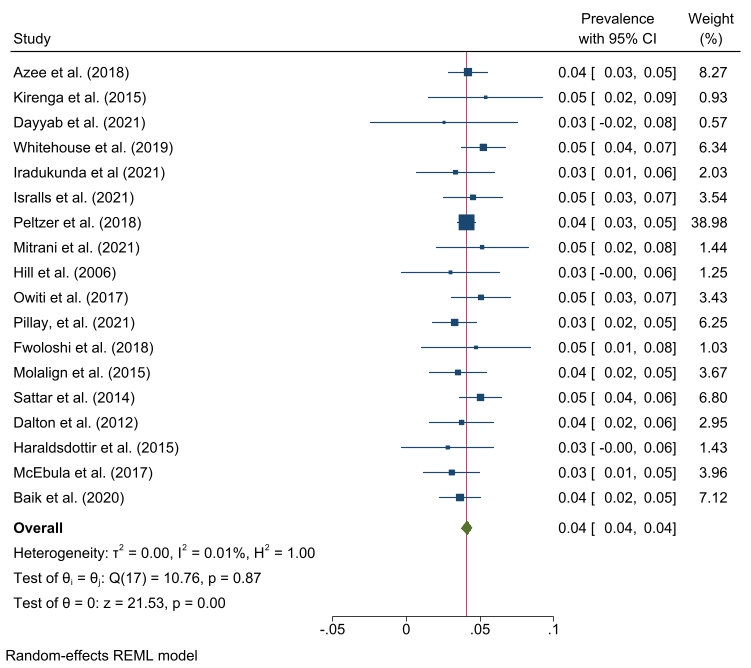


## Figure 5: Sub-analysis for prevalence of obesity in people with TB among studies within the funnel plot


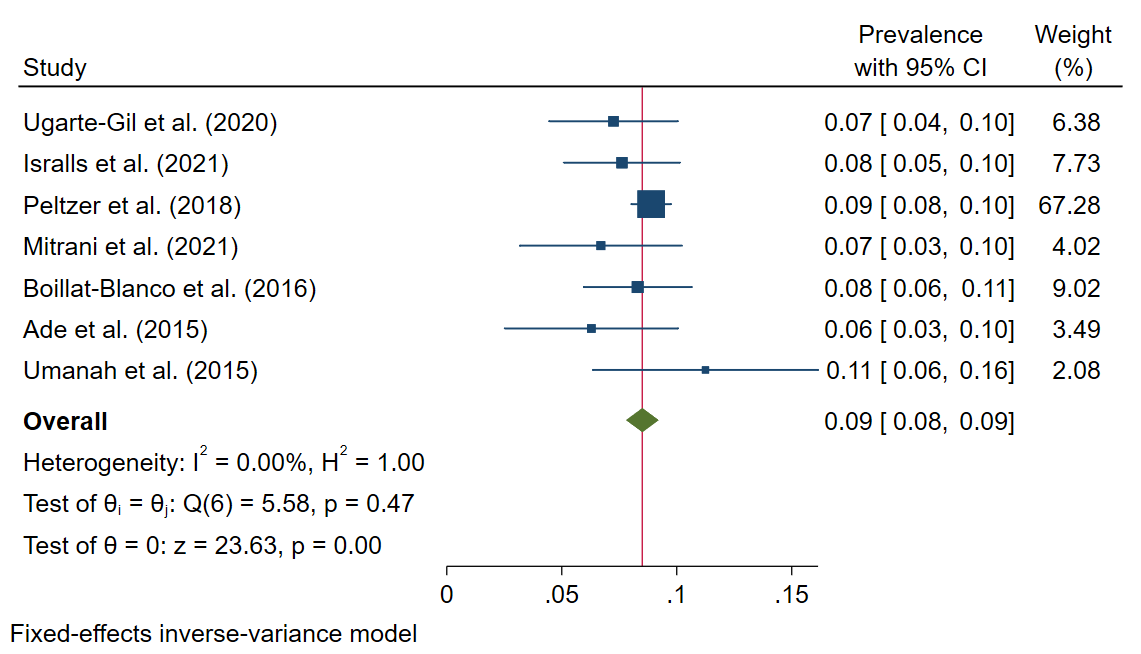


## Figure 6: Sub-analysis for prevalence of hypertension in people with TB among studies within the funnel plot

# Figure 7: PRISMA flow diagram

Studies identified through database search
(n = 1,238)

Duplicates removed at initial stage
(n = 248)

Identification

Screening

Records screened
(n = 990)

Records assessed for eligibility
(n = 143)

Studies included in qualitative synthesis and metanalysis
(n = 110)

Studies included in meta-analysis for alcohol use
(n = 67)

Studies included in meta-analysis for smoking
(n = 79)

Studies included in meta-analysis for DM
(n = 51)

Studies included in meta-analysis for hypertension
(n = 19)

Studies included in meta-analysis for obesity
(n = 18)

Studies included in meta-analysis for hazardous alcohol use
(n = 30)

Eligibility

Included

Records excluded
(n = 847)

# Figure 8: Funnel plots for assessing publication bias for prevalence of cardiovascular risk factors in people with active TB in Africa


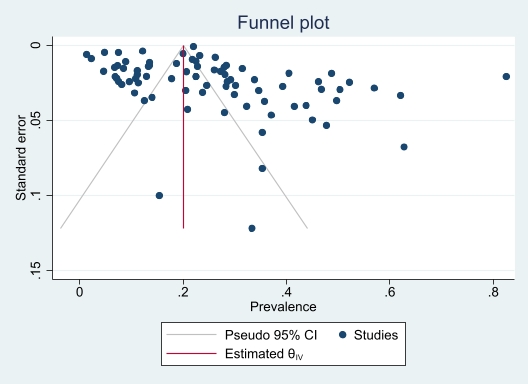


**Smoking**


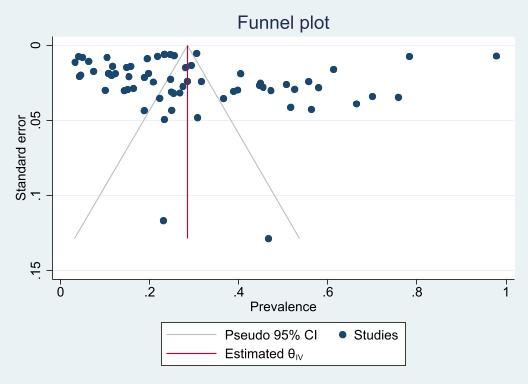


**Current alcohol use**


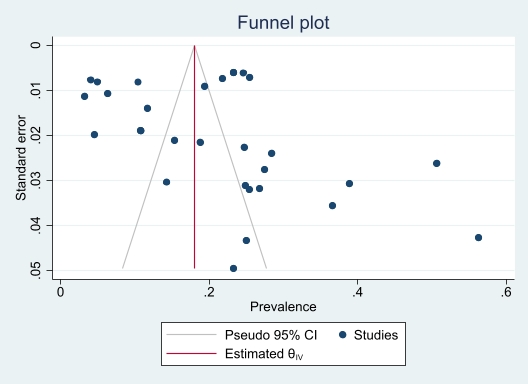


**Hazardous alcohol use**


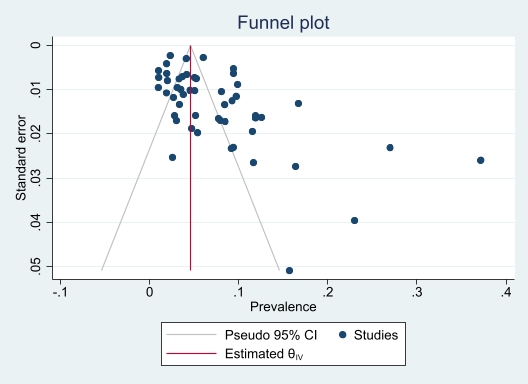


**Diabetes mellitus**


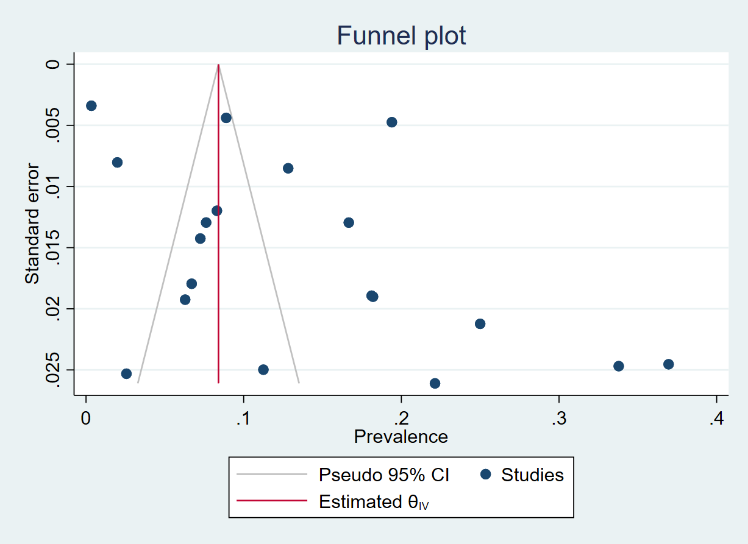


**Hypertension**


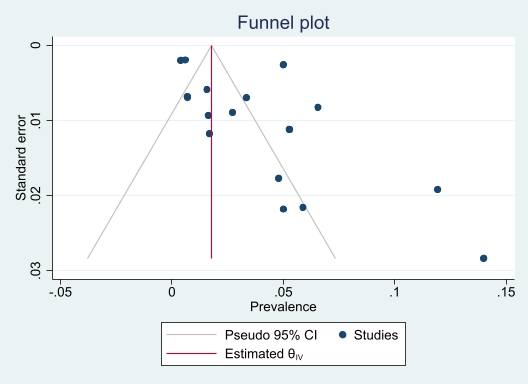


**Obesity**

# Table 6: Risk of bias scores using Hoy and colleagues’ tool [111]

The mean (standard deviation) score was 7.9 (1.4). Majority of studies (63.6%, 70/110) had a score of ≥ 8.

| **Authors** | **Was the study’s target population a close representation of the population in relation to relevant variables?** | **Was the sampling frame a true or close representation of the target population?** | **Was some form of random selection used to select the sample, OR was a census undertaken?** | **Was the likelihood of nonresponse bias minimal?** | **Were data collected directly from the subjects (as opposed to a proxy)?** | **Was an acceptable case definition of the cardiovascular risk factor used in the study?** | **Was the study method that diagnosed the cardiovascular risk factor shown to have validity and reliability?** | **Was the same mode of data collection used for all subjects?** | **Was the length of the shortest prevalence period for the parameter of interest appropriate?** | **Were the numerator(s) and denominator(s) for the parameter of interest appropriate and clearly reported?** | **Total** |
| --- | --- | --- | --- | --- | --- | --- | --- | --- | --- | --- | --- |
| Asante-Poku, et al. (2019) | 1 | 1 | 1 | 1 | 1 | 1 | 1 | 1 | 1 | 1 | 10 |
| Appiah, et al (2021) | 1 | 1 | 1 | 1 | 1 | 1 | 1 | 1 | 1 | 1 | 10 |
| Tiamiyu, et al. (2020) | 1 | 1 | 1 | 1 | 1 | 1 | 1 | 1 | 1 | 1 | 10 |
| Wanyonyi, et al. (2017) | 1 | 1 | 1 | 1 | 1 | 1 | 1 | 1 | 1 | 1 | 10 |
| Soboka, et al. (2021) | 1 | 1 | 1 | 1 | 1 | 1 | 1 | 1 | 1 | 1 | 10 |
| Seegert, et al. (2021) | 1 | 1 | 1 | 1 | 1 | 1 | 1 | 1 | 1 | 1 | 10 |
| Faurholt-Jepsen, et al. (2011) | 1 | 1 | 1 | 1 | 1 | 1 | 1 | 1 | 1 | 1 | 10 |
| Faurholt-Jepsen, et al. (2012) | 1 | 1 | 1 | 1 | 1 | 1 | 1 | 1 | 1 | 1 | 10 |
| Hayes-Larson, et al. (2017) | 1 | 1 | 1 | 1 | 1 | 1 | 1 | 1 | 1 | 1 | 10 |
| Churchyard, et al. (2010) | 1 | 1 | 1 | 1 | 1 | 1 | 1 | 1 | 1 | 1 | 10 |
| Segafredo, et al. (2019) | 1 | 1 | 1 | 1 | 1 | 1 | 1 | 1 | 1 | 1 | 10 |
| Tola, et al. (2015) | 1 | 1 | 1 | 1 | 1 | 1 | 1 | 1 | 1 | 1 | 10 |
| Peltzer, et al. (2013) | 1 | 1 | 1 | 1 | 1 | 1 | 1 | 1 | 1 | 1 | 10 |
| Peltzer, et al. (2013) | 1 | 1 | 1 | 1 | 1 | 1 | 1 | 1 | 1 | 1 | 10 |
| Brunet, et al. (2011) | 1 | 1 | 1 | 1 | 1 | 1 | 1 | 1 | 1 | 1 | 10 |
| Zetola, et al. (2012) | 1 | 1 | 1 | 1 | 1 | 1 | 1 | 1 | 1 | 1 | 10 |
| Stracker, et al. (2019) | 1 | 1 | 1 | 1 | 1 | 1 | 1 | 1 | 1 | 1 | 10 |
| Tulu, et al. (2021) | 1 | 1 | 1 | 1 | 1 | 1 | 1 | 1 | 1 | 1 | 10 |
| Muture, et al. (2011) | 1 | 1 | 1 | 1 | 1 | 1 | 0 | 1 | 1 | 1 | 9 |
| Oumer, et al. (2021) | 1 | 1 | 1 | 1 | 1 | 1 | 0 | 1 | 1 | 1 | 9 |
| Ugarte-Gil, et al. (2020) | 1 | 1 | 0 | 1 | 1 | 1 | 1 | 1 | 1 | 1 | 9 |
| Kibirige, et al. (2013) | 1 | 1 | 0 | 1 | 1 | 1 | 1 | 1 | 1 | 1 | 9 |
| Pizzol, et al. (2017) | 1 | 0 | 1 | 1 | 1 | 1 | 1 | 1 | 1 | 1 | 9 |
| Whitehouse, et al. (2019) | 1 | 1 | 1 | 1 | 0 | 1 | 1 | 1 | 1 | 1 | 9 |
| Matseke, et al. (2012) | 0 | 1 | 1 | 1 | 1 | 1 | 1 | 1 | 1 | 1 | 9 |
| Otwombe, et al. (2013) | 1 | 1 | 1 | 1 | 1 | 1 | 0 | 1 | 1 | 1 | 9 |
| Peltzer, et al. (2018) | 1 | 1 | 1 | 1 | 1 | 1 | 0 | 1 | 1 | 1 | 9 |
| Kubjane, et al. (2020) | 1 | 1 | 0 | 1 | 1 | 1 | 1 | 1 | 1 | 1 | 9 |
| Boillat-Blanco, et al. (2016) | 1 | 1 | 0 | 1 | 1 | 1 | 1 | 1 | 1 | 1 | 9 |
| Ekeke, et al. (2017) | 1 | 1 | 1 | 1 | 1 | 1 | 0 | 1 | 1 | 1 | 9 |
| Naidoo, et al. (2013) | 1 | 1 | 0 | 1 | 1 | 1 | 1 | 1 | 1 | 1 | 9 |
| Owiti, et al. (2017) | 1 | 1 | 0 | 1 | 1 | 1 | 1 | 1 | 1 | 1 | 9 |
| O'Connell, et al. (2013) | 1 | 1 | 1 | 0 | 1 | 1 | 1 | 1 | 1 | 1 | 9 |
| Bailey, et al. (2016) | 1 | 1 | 1 | 0 | 1 | 1 | 1 | 1 | 1 | 1 | 9 |
| Nagu, et al. (2017) | 1 | 1 | 1 | 1 | 1 | 1 | 0 | 1 | 1 | 1 | 9 |
| Oni, et al. (2017) | 0 | 1 | 0 | 1 | 1 | 1 | 1 | 1 | 1 | 1 | 9 |
| Shangase, et al. (2018) | 1 | 1 | 1 | 0 | 1 | 1 | 1 | 1 | 1 | 1 | 9 |
| Soboka, et al (2021) | 1 | 1 | 0 | 1 | 1 | 1 | 1 | 1 | 1 | 1 | 9 |
| Van't Hoog, et al. (2013) | 1 | 1 | 1 | 0 | 1 | 1 | 0 | 1 | 1 | 1 | 8 |
| Kirenga, et al. (2015) | 1 | 1 | 0 | 0 | 1 | 1 | 1 | 1 | 1 | 1 | 8 |
| t Veld, et al. (2018) | 0 | 0 | 1 | 1 | 1 | 1 | 1 | 1 | 1 | 1 | 8 |
| Ayeni, et al (2021) | 1 | 1 | 0 | 0 | 1 | 1 | 1 | 1 | 1 | 1 | 8 |
| Talbot, et al. (2002) | 1 | 1 | 0 | 1 | 1 | 1 | 0 | 1 | 1 | 1 | 8 |
| Yorke, et al. (2018) | 1 | 1 | 0 | 0 | 1 | 1 | 1 | 1 | 1 | 1 | 8 |
| Louwagie et al. (2013) | 0 | 1 | 1 | 1 | 0 | 1 | 1 | 1 | 1 | 1 | 8 |
| Iweama et al. (2021) | 1 | 1 | 1 | 0 | 1 | 1 | 0 | 1 | 1 | 1 | 8 |
| Nsonga, et al. (2019) | 0 | 1 | 1 | 0 | 1 | 1 | 1 | 1 | 1 | 1 | 8 |
| Gunasekera, et al. (2020) | 1 | 1 | 1 | 0 | 1 | 1 | 0 | 1 | 1 | 1 | 8 |
| Mabula et al. (2021) | 0 | 1 | 1 | 0 | 1 | 1 | 1 | 1 | 1 | 1 | 8 |
| Iradukunda, et al (2021) | 1 | 1 | 1 | 1 | 1 | 0 | 0 | 1 | 1 | 1 | 8 |
| Zetola, et al. (2021) | 1 | 1 | 1 | 0 | 1 | 1 | 0 | 1 | 1 | 1 | 8 |
| Petersen, et al (2021) | 1 | 1 | 1 | 0 | 1 | 1 | 0 | 1 | 1 | 1 | 8 |
| Wekunda, et al (2021) | 1 | 1 | 1 | 0 | 1 | 1 | 0 | 1 | 1 | 1 | 8 |
| Kirubi, et al (2021) | 1 | 1 | 1 | 1 | 1 | 0 | 0 | 1 | 1 | 1 | 8 |
| Erisa, et al. (2021) | 1 | 1 | 0 | 0 | 1 | 1 | 1 | 1 | 1 | 1 | 8 |
| Pillay, et al (2021) | 1 | 1 | 0 | 0 | 0 | 0 | 0 | 1 | 1 | 1 | 5 |
| Tekie Desta, et al. (2018) | 1 | 1 | 1 | 1 | 1 | 0 | 0 | 1 | 1 | 1 | 8 |
| Lawson, et al. (2017) | 1 | 1 | 0 | 0 | 1 | 1 | 1 | 1 | 1 | 1 | 8 |
| Kassa, et al (2021) | 1 | 1 | 1 | 1 | 0 | 1 | 0 | 1 | 1 | 1 | 8 |
| Munseri, et al. (2019) | 1 | 1 | 0 | 1 | 1 | 1 | 0 | 1 | 1 | 1 | 8 |
| Sonnenberg, et al. (2000) | 1 | 0 | 1 | 1 | 1 | 1 | 0 | 1 | 1 | 1 | 8 |
| Mwiru, et al. (2017) | 1 | 1 | 0 | 0 | 1 | 1 | 1 | 1 | 1 | 1 | 8 |
| Diande, et al. (2019) | 1 | 1 | 0 | 1 | 1 | 1 | 0 | 1 | 1 | 1 | 8 |
| Fwoloshi, et al. (2018) | 1 | 1 | 0 | 1 | 1 | 1 | 0 | 1 | 1 | 1 | 8 |
| Sattar, et al. (2014) | 1 | 1 | 1 | 0 | 1 | 1 | 0 | 1 | 1 | 1 | 8 |
| Haraldsdottir, et al. (2015) | 1 | 1 | 1 | 0 | 1 | 1 | 0 | 1 | 1 | 1 | 8 |
| Kombila, et al. (2018) | 1 | 1 | 0 | 1 | 1 | 1 | 0 | 1 | 1 | 1 | 8 |
| McEbula, et al. (2017) | 1 | 1 | 0 | 1 | 1 | 1 | 0 | 1 | 1 | 1 | 8 |
| Araia, et a. (2021) | 1 | 1 | 1 | 1 | 0 | 0 | 1 | 1 | 1 | 1 | 8 |
| Baik, et al. (2020) | 1 | 1 | 1 | 1 | 1 | 0 | 0 | 1 | 1 | 1 | 8 |
| Adegbite, et al. (2020) | 1 | 1 | 0 | 0 | 1 | 1 | 1 | 1 |  | 1 | 8 |
| Azee, et al. (2018) | 1 | 1 | 1 | 1 | 0 | 0 | 0 | 1 | 1 | 1 | 7 |
| Balkissou, et al. (2016) | 1 | 1 | 0 | 1 | 1 | 0 | 0 | 1 | 1 | 1 | 7 |
| Isralls, et al. | 1 | 1 | 1 | 1 | 0 | 0 | 0 | 1 | 1 | 1 | 7 |
| Farghaly, et al. (2021) | 1 | 1 | 0 | 0 | 1 | 1 | 0 | 1 | 1 | 1 | 7 |
| Musuenge, et al. (2020) | 1 | 1 | 0 | 0 | 1 | 1 | 0 | 1 | 1 | 1 | 7 |
| Lam, et al. (2013) | 1 | 0 | 0 | 1 | 1 | 1 | 1 | 1 | 1 | 1 | 7 |
| Mollel et al. (2017) | 1 | 1 | 1 | 1 | 0 | 0 | 0 | 1 | 1 | 1 | 7 |
| Mitrani, et al. (2021) | 1 | 1 | 1 | 1 | 0 | 0 | 0 | 1 | 1 | 1 | 7 |
| Gebrehiwet, et al. (2019) | 1 | 1 | 0 | 1 | 1 | 0 | 0 | 1 | 1 | 1 | 7 |
| Mulisa, et al. (2015) | 1 | 1 | 0 | 1 | 1 | 0 | 0 | 1 | 1 | 1 | 7 |
| Dayyab, et al. (2021) | 1 | 1 | 0 | 1 | 1 | 0 | 0 | 1 | 1 | 1 | 7 |
| Racil, et al. (2012) | 1 | 1 | 1 | 1 | 0 | 0 | 0 | 1 | 1 | 1 | 7 |
| Adetifa, et al. (2017) | 1 | 1 | 0 | 0 | 1 | 1 | 0 | 1 | 1 | 1 | 7 |
| Murrison, et al. (2016) | 1 | 0 | 0 | 0 | 1 | 1 | 1 | 1 | 1 | 1 | 7 |
| Ali, et al. (2019) | 1 | 1 | 1 | 0 | 1 | 0 | 0 | 1 | 1 | 1 | 7 |
| Magee, et al. (2017) | 1 | 1 | 0 | 0 | 1 | 1 | 0 | 1 | 1 | 1 | 7 |
| Berkowitz, et al. (2018) | 1 | 0 | 0 | 1 | 1 | 1 | 0 | 1 | 1 | 1 | 7 |
| Hill, et al. (2006) | 1 | 1 | 0 | 0 | 1 | 1 | 0 | 1 | 1 | 1 | 7 |
| Ncube, et al. (2019) | 1 | 1 | 0 | 0 | 0 | 1 | 1 | 1 | 1 | 1 | 7 |
| Ade, et al. (2015) | 1 | 1 | 0 | 0 | 0 | 1 | 1 | 1 | 1 | 1 | 7 |
| Kufa, et al. (2016) | 1 | 0 | 0 | 1 | 1 | 1 | 0 | 1 | 1 |  | 7 |
| Bhana, et al. (2017) | 0 | 0 | 0 | 0 | 1 | 1 | 1 | 1 | 1 | 1 | 6 |
| Assal, et al (2021) | 1 | 0 | 0 | 0 | 1 | 1 | 0 | 1 | 1 | 1 | 6 |
| Kebede, et al. (2021) | 1 | 0 | 1 | 1 | 0 | 0 | 0 | 1 | 1 | 1 | 6 |
| Conradie, et al. (2014) | 1 | 1 | 0 | 1 | 1 | 0 | 0 | 1 | 1 | 0 | 6 |
| Watermeyer et al. (2018) | 1 | 0 | 1 | 1 | 0 | 0 | 0 | 1 | 1 | 1 | 6 |
| Mburu, et al. (2018) | 1 | 1 | 0 | 0 | 1 | 0 | 0 | 1 | 1 | 1 | 6 |
| Wessels, et al. (2019) | 0 | 1 | 0 | 0 | 1 | 1 | 0 | 1 | 1 | 1 | 6 |
| Ngosa et al. (2016) | 1 | 0 | 0 | 1 | 0 | 1 | 0 | 1 | 1 | 1 | 6 |
| Nyasulu, et al. (2015) | 1 | 0 | 1 | 1 | 0 | 0 | 0 | 1 | 1 | 1 | 6 |
| Molalign, et al. (2015) | 1 | 1 | 1 | 1 | 0 | 0 | 0 | 1 | 1 | 1 | 6 |
| Dalton, et al. (2012) | 1 | 1 | 1 | 0 | 0 | 0 | 1 | 0 | 1 | 1 | 6 |
| Oni, et al. (2015) | 0 | 1 | 1 | 1 | 0 | 0 | 0 | 1 | 1 | 1 | 6 |
| Umanah, et al. (2015) | 1 | 0 | 1 | 1 | 0 | 0 | 0 | 1 | 1 | 1 | 6 |
| Wotale, et al (2021) | 1 | 1 | 1 | 0 | 0 | 0 | 0 | 1 | 1 | 1 | 6 |
| Sitas, et al. (2004) | 1 | 0 | 1 | 1 | 0 | 0 | 0 | 1 | 1 | 1 | 5 |
| Deetlefs, et al. (2012) | 1 | 0 | 1 | 0 | 0 | 0 | 0 | 1 | 1 | 1 | 5 |
| Kootbodien, et al. (2018) | 0 | 0 | 1 | 1 | 0 | 0 | 0 | 1 | 1 | 1 | 5 |
| Sidamo, et al (2021) | 1 | 1 | 0 | 0 | 0 | 0 | 0 | 1 | 1 | 1 | 5 |

# Table 7: Studies excluded after full text review

| **Authors** | **Reason for exclusion** |
| --- | --- |
| 1. Harling, et al. (2008) [112] | Did not provide data on proportions/prevalence of smoking and alcohol use among people with tuberculosis |
| 1. Webb, et al. (2009)[113] | The study focused on TB in diabetes and had < 10 participants with TB |
| 1. Badawi, et al. (2014)[114] | The study was an ecological modelling study |
| 1. Philips, et al. (2017)[115] | Individuals with obesity, diabetes, HIV, metabolic syndrome were specifically excluded |
| 1. Harries, et al. (2011)[116] | Narrative review |
| 1. Van Rensburg, et al. (2020)[117] | Narrative review |
| 1. Riza, et al. (2014)[118] | Narrative review |
| 1. Renzaho (2015)[119] | Narrative review |
| 1. Marais, et al. (2013)[120] | Narrative review |
| 1. Njuguna, et al. (2018) [121] | Narrative review |
| 1. Germe, et al (2017) [122] | Full text article could not be located |
| 1. Bartlett, et al. (2020)[123] | < 10 participants |
| 1. Harries, et al (2010) [124] | Opinion article |
| 1. Harries, et al. (2019) [125] | Opinion article |
| 1. Okunola, et al. (2011) [126] | No relevant data on TB and cardiovascular risk factors |
| 1. Young, et al (2009) [127] | Narrative review |
| 1. Louw, et al (2012) [128] | Same population as in Peltzer, et al (2013) [70] |
| 1. Louw, et al (2016) [129] | Same population as in Peltzer, et al. (2013) [85] |
| 1. Peltzer (2014) [130] | Same population as in Peltzer, et al (2013) [70] |
| 1. Peltzer (2016) [131] | Same population as in Peltzer, et al (2013) [70] |
| 1. Peltzer, et al. (2013) [132] | Same population as in Peltzer, et al (2013) [70] |
| 1. Peltzer, et al (2014) [133] | Same population as in Peltzer, et al. (2013) [85] |
| 1. Peltzer, et al. (2012) [134] | Same population as in Peltzer, et al (2013) [70] |
| 1. Peltzer, et al. (2012) [135] | Same population as in Peltzer, et al. (2013) [85] |
| 1. Peltzer, et al. (2012) [136] | Same population as in Peltzer, et al (2013) [70] |
| 1. Magu, et al. (2016) [137] | Full text article could not be located |
| 1. Wachinou, et al (2021) [138] | Did not provide data on proportions/prevalence of CVD risk factors in the people with TB |
| 1. Hopkins, et al. (2021) [139] | Did not provide data on proportions/prevalence of CVD risk factors in the people with TB |
| 1. Mzembe, at al (2021) [140] | Study was among people with *Mtb* infection and not active TB |
| 1. Mohamed, et al (2021) [141] | Did not provide data on proportions/prevalence of CVD risk factors in the people with TB |
| 1. Alisjahbana, et al. (2021) [142] | Did not provide data on proportions/prevalence of CVD risk factors in the people with TB in South Africa |
| 1. Wong, et al. (2021) [143] | Did not provide data on proportions/prevalence of CVD risk factors in the people with TB |
| 1. Henry, et al (2021) [144] | Did not provide data on proportions/prevalence of CVD risk factors in the people with TB in the African countries involved. |

# Table 8: Meta Regression analyses for prevalence of cardiovascular risk factors among people with active TB in Africa

| **Variable** | **Coefficient** | **95% CI** | **p-value** |
| --- | --- | --- | --- |
| **HYPERTENSION** |  |  |  |
| **Region** |  |  |  |
| Eastern Africa | 1.00 |  |  |
| Southern Africa | -0.08 | -0.19 - 0.02 | 0.116 |
| West Africa | -0.16 | -0.29 - -0.03 | 0.013 |
| **Diagnostic criteria** | |  |  |
| Unknown | 1.00 |  |  |
| Blood pressure | 0.09 | -0.01 - 0.18 | 0.066 |
| Medication | 0.13 | -0.02 - 0.28 | 0.086 |
| **Study design** |  |  |  |
| Case-control | 1.00 |  |  |
| Cohort | 0.06 | -0.16 - 0.29 | 0.599 |
| Cross-sectional | 0.06 | -0.16 - 0.27 | 0.606 |
| **Risk of bias** |  |  |  |
| Low | 1.00 |  |  |
| Moderate | -0.01 | -0.1 - 0.09 | 0.847 |
| **TB drug sensitivity** | |  |  |
| Both resistant and susceptible | | |  |
| Drug resistant | 0.01 | -0.16 - 0.18 | 0.926 |
| Unknown | 0.02 | -0.14 - 0.18 | 0.787 |
| **DIABETES MELLITUS** | |  |  |
| **Diagnostic criteria** | |  |  |
| Standard* | 1.00 |  |  |
| Self report | -0.04 | -0.08 - 0.01 | 0.137 |
| Unknown | -0.03 | -0.07 - 0.01 | 0.158 |
| **Region** |  |  |  |
| Eastern Africa | 1.00 |  |  |
| Northern Africa | 0.07 | -0.03 - 0.18 | 0.173 |
| Southern Africa | -0.03 | -0.07 - 0.01 | 0.165 |
| West Africa | -0.02 | -0.07 - 0.02 | 0.308 |
| **Study design** |  |  |  |
| Case-control | 1.00 |  |  |
| Cohort | -0.02 | -0.1 - 0.06 | 0.681 |
| Cross sectional nested in RCT | -0.03 | -0.12 - 0.07 | 0.575 |
| Observational cross sectional | 0.00 | -0.07 - 0.07 | 0.946 |
| **Risk of bias** |  |  |  |
| Low | 1.00 |  |  |
| Moderate | -0.01 | -0.05 - 0.03 | 0.520 |
| **TB drug sensitivity** | |  |  |
| Both resistant and susceptible | 1.00 |  |  |
| Drug resistant | 0.00 | -0.08 - 0.07 | 0.897 |
| Sensitive TB | -0.06 | -0.2 - 0.08 | 0.425 |
| Unknown | 0.00 | -0.06 - 0.06 | 0.958 |
| **OBESITY** |  |  |  |
| **Region** |  |  |  |
| Eastern Africa | 1.00 |  |  |
| Southern Africa | 0.03 | 0 - 0.07 | 0.082 |
| West Africa | 0.02 | -0.02 - 0.06 | 0.359 |
| **Study design** |  |  |  |
| Cohort | 1.00 |  |  |
| Cross sectional nested in RCT | 0.01 | -0.06 - 0.07 | 0.814 |
| Observational cross sectional | 0.01 | -0.04 - 0.06 | 0.612 |
| **Risk of bias** |  |  |  |
| Low | 1.00 |  |  |
| Moderate | -0.02 | -0.05 - 0.01 | 0.118 |
| **TB drug sensitivity** | |  |  |
| **Both resistant and susceptible** | 1.00 |  |  |
| Drug resistant | 0.01 | -0.08 - 0.1 | 0.778 |
| Unknown | -0.02 | -0.08 - 0.05 | 0.610 |
| **CURRENT ALCOHOL USE** | |  |  |
| **Type of drinking** | |  |  |
| Hazardous alcohol use | 1.00 |  |  |
| Non-hazardous alcohol use | 0.16 | 0.07 - 0.25 | 0.001 |
| **Region** |  |  |  |
| Eastern Africa | 1.00 |  |  |
| Northern Africa | -0.01 | -0.31 - 0.3 | 0.962 |
| Southern Africa | -0.03 | -0.15 - 0.08 | 0.586 |
| West Africa | -0.02 | -0.17 - 0.12 | 0.739 |
| **Study design** |  |  |  |
| Case-control | 1.00 |  |  |
| Cohort | 0.01 | -0.17 - 0.19 | 0.927 |
| Cross sectional nested in RCT | -0.06 | -0.34 - 0.21 | 0.657 |
| Observational cross sectional | 0.04 | -0.12 - 0.19 | 0.634 |
| **Risk of bias** |  |  |  |
| Low | 1.00 |  |  |
| Moderate | 0.01 | -0.09 - 0.11 | 0.828 |
| **TB drug sensitivity** | |  |  |
| **Both resistant and susceptible** | 1.00 |  |  |
| Drug resistant | -0.08 | -0.26 - 0.09 | 0.355 |
| Susceptible | -0.25 | -0.67 - 0.17 | 0.250 |
| Unknown | -0.06 | -0.19 - 0.07 | 0.358 |
| **HAZARDOUS ALCOHOL USE** | |  |  |
| **Diagnostic criteria** | |  |  |
| AUDIT | 1.00 |  |  |
| Daily use | -0.10 | -0.23 - 0.02 | 0.106 |
| Others | 0.05 | -0.04 - 0.15 | 0.285 |
| **Region** |  |  |  |
| Eastern Africa | 1.00 |  |  |
| Northern Africa | 0.09 | -0.11 - 0.29 | 0.368 |
| Southern Africa | -0.01 | -0.11 - 0.1 | 0.919 |
| West Africa | -0.09 | -0.26 - 0.08 | 0.318 |
| **Study design** |  |  |  |
| Case-control | 1.00 |  |  |
| Cohort | -0.10 | -0.26 - 0.07 | 0.242 |
| Observational cross sectional | 0.01 | -0.12 - 0.15 | 0.842 |
| **Risk of bias** |  |  |  |
| Low | 1.00 |  |  |
| Moderate | 0.00 | -0.09 - 0.1 | 0.917 |
| **TB drug sensitivity** | |  |  |
| Both resistant and susceptible | 1.00 |  |  |
| Drug resistant | -0.14 | -0.31 - 0.03 | 0.114 |
| Susceptible | -0.18 | -0.44 - 0.08 | 0.178 |
| Unknown | -0.09 | -0.2 - 0.03 | 0.142 |
| **SMOKING** |  |  |  |
| **Region** |  |  |  |
| Eastern Africa | 1.00 |  |  |
| Northern Africa | 0.24 | 0.08 - 0.41 | 0.004 |
| Southern Africa | 0.09 | 0.01 - 0.17 | 0.024 |
| West Africa | -0.03 | -0.13 - 0.06 | 0.511 |
| **Study design** |  |  |  |
| Case-control | 1.00 |  |  |
| Cohort | -0.13 | -0.26 - 0.01 | 0.069 |
| Cross sectional nested in RCT | -0.07 | -0.23 - 0.1 | 0.416 |
| Observational cross sectional | -0.13 | -0.24 - -0.02 | 0.025 |
| Randomised controlled trial | 0.13 | -0.2 - 0.45 | 0.448 |
| **Risk of bias** |  |  |  |
| Low | 1.00 |  |  |
| Moderate | 0.05 | -0.03 - 0.13 | 0.252 |
| **TB drug sensitivity** | |  |  |
| Both resistant and susceptible | 1.00 |  |  |
| Drug resistant | 0.06 | -0.08 - 0.2 | 0.410 |
| Unknown | 0.06 | -0.05 - 0.16 | 0.278 |
| **Diagnostic criteria** | |  |  |
| Current smoker | 1.00 |  |  |
| Ever smoked | 0.02 | -0.07 - 0.11 | 0.643 |
| Lifetime cigarettes >100 | -0.14 | -0.33 - 0.05 | 0.160 |
| Others | 0.06 | -0.09 - 0.22 | 0.421 |
| Unknown | -0.04 | -0.13 - 0.06 | 0.436 |

*elevated HbA1c ≥ 6.5% or fasting blood sugar (FBS) ≥126mg/dl or 2hour plasma glucose of ≥200mg/dl after oral glucose tolerance test or random blood sugar (RBS) ≥11.1 mmol/l with symptoms of DM or use of DM medication

# Table 9: Prevalence of cardiovascular risk factors among people with active TB in Africa after non-parametric trim and fill.

| **Studies** | **Prevalence** | **LCI** | **UCI** | **Observed** | **Imputed** |
| --- | --- | --- | --- | --- | --- |
| **Hypertension** |  |  |  | 19 | 0 |
| Observed | 13.8% | 9.2% | 18.4% |  |  |
| Observed + Imputed | 13.8% | 9.2% | 18.4% |  |  |
| **Diabetes mellitus** |  |  |  |  |  |
| Observed | 7.5% | 5.7% | 9.2% | 51 | 0 |
| Observed + Imputed | 7.5% | 5.7% | 9.2% |  |  |
| **Obesity** |  |  |  |  |  |
| Observed | 1.8% | 1.6% | 2.0% | 19 | 4 |
| Observed + Imputed | 1.7% | 1.5% | 1.9% |  |  |
| **Current alcohol use** |  |  |  | 66 | 9 |
| Observed | 29.9% | 24.9% | 34.9% |  |  |
| Observed + Imputed | 33.7% | 28.8% | 38.7% |  |  |
| **Hazardous alcohol use** | |  |  | 34 | 0 |
| Observed | 18.0% | 17.6% | 18.4% |  |  |
| Observed + Imputed | 18.0% | 17.6% | 18.4% |  |  |
| **Smoking** |  |  |  | 79 | 0 |
| Observed | 25.6% | 22.0% | 29.2% |  |  |
| Observed + Imputed | 25.6% | 22.0% | 29.2% |  |  |

LCI – lower confidence interval, UCI – upper confidence interval

# Figure 9: Contour-enhanced funnel plots after trim and fill for assessing publication bias for prevalence of cardiovascular risk factors in people with active TB in Africas


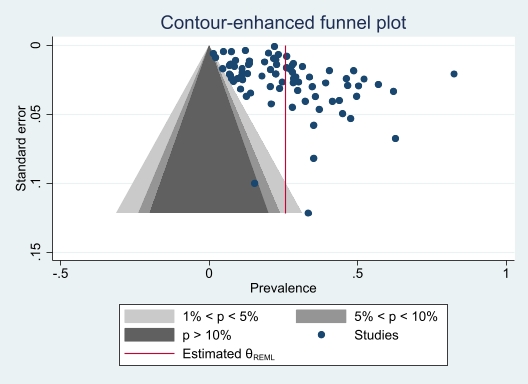


**Smoking**


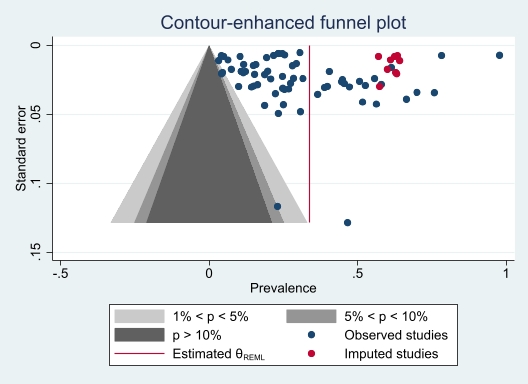


**Current alcohol use**


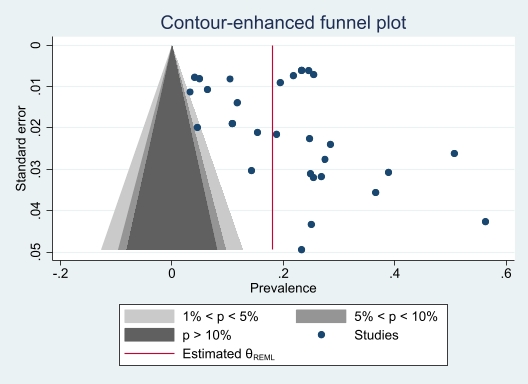


**Hazardous alcohol use**


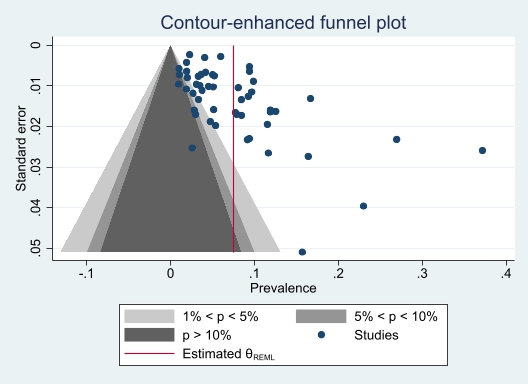


**Diabetes mellitus**


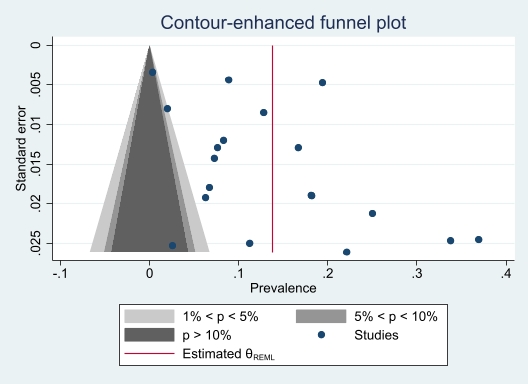


**Hypertension**


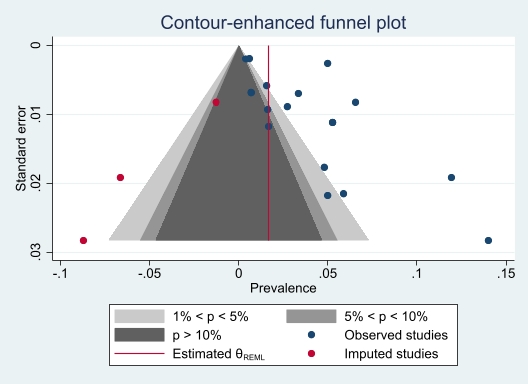


**Obesity**

# Table 10: Search Strategy

|  | **Medline** | **Embase** | **Cochrane Library** | **Web of Science** |
| --- | --- | --- | --- | --- |
| **Search string** | Ovid MEDLINE(R) ALL <1946 to June 13, 2022>  1 (tuberculosis or TB or PTB).ti,ab. 232375 2 Tuberculosis/ 110626 3 1 or 2 262702 4 ((hypertension or diabetes or pre-diabetes or cardiovascular or metabolic syndrome or hyperlipid?emia* or dyslipid?emia* or cholesterol or hypercholesterolemia or low density lipoprotein or triglycerides) and (risk* or alcohol or smoking or smoke* or cigarette* or overweight or obese or obesity or physical inactivity)).ti,ab. 559477 5 Hypertension/ or diabetes mellitus/ or Obesity/ or heart disease risk factors/ or hyperlipidemias/ or metabolic syndrome/ or dyslipidemias/ or Alcohol Drinking/ or smoking/ 790988 6 (Algeria or Angola or Benin or Botswana or "Burkina Faso" or Burundi or Cameroon or "Cape Verde" or "Central African Republic" or Chad or Comoros or "Democratic Republic of Congo" or Djibouti or Egypt or "Equatorial Guinea" or Eritrea or Ethiopia or Gabon or Gambia or Ghana or Guinea or "Guinea Bissau" or "Ivory Coast" or "Cote d’Ivoire" or Kenya or Lesotho or Liberia or Libya or Libya or Madagascar or Malawi or Mali or Mauritania or Mauritius or Morocco or Mozambique or Namibia or Niger or Nigeria or Rwanda or "Sao Tome" or Senegal or Seychelles or "Sierra Leone" or Somalia or "South Africa" or "South Sudan" or Sudan or Swaziland or Tanzania or Togo or Tunisia or Uganda or Zaire or Zambia or Zimbabwe or "Central Africa" or "West Africa" or "Western Africa" or "East Africa" or "Eastern Africa" or "North Africa" or "Northern Africa" or "Southern Africa" or "sub Saharan Africa" or "subsaharan Africa" or Africa).ti,ab. 437462 7 4 or 5 1179511 8 (prevalen* or incidence* or burden*).ti,ab. or Prevalence/ or Incidence/ 2053676 9 3 and 6 and 7 and 8 293 10 limit 9 to yr="1860 - 2020" 244 11 limit 9 to yr="2021" 40 | Embase <1974 to 2022 June 13>  1 (tuberculosis or TB or PTB).ti,ab. 231400 2 Tuberculosis/ 121045 3 1 or 2 262047 4 ((hypertension or diabetes or pre-diabetes or cardiovascular or metabolic syndrome or hyperlipid?emia* or dyslipid?emia* or cholesterol or hypercholesterolemia or triglycerides or low density lipoprotein) and (risk* or alcohol or smoking or smoke* or cigarette* or overweight or obese or obesity or physical inactivity)).ti,ab. 893656 5 Hypertension/ or diabetes mellitus/ or Obesity/ or heart disease risk factor/ or hyperlipidemias/ or metabolic syndrome/ or dyslipidemias/ or alcohol consumption/ or smoking/ 1881252 6 (Algeria or Angola or Benin or Botswana or "Burkina Faso" or Burundi or Cameroon or "Cape Verde" or "Central African Republic" or Chad or Comoros or "Democratic Republic of Congo" or Djibouti or Egypt or "Equatorial Guinea" or Eritrea or Ethiopia or Gabon or Gambia or Ghana or Guinea or "Guinea Bissau" or "Ivory Coast" or "Cote d’Ivoire" or Kenya or Lesotho or Liberia or Libya or Libya or Madagascar or Malawi or Mali or Mauritania or Mauritius or Morocco or Mozambique or Namibia or Niger or Nigeria or Rwanda or "Sao Tome" or Senegal or Seychelles or "Sierra Leone" or Somalia or "South Africa" or "South Sudan" or Sudan or Swaziland or Tanzania or Togo or Tunisia or Uganda or Zaire or Zambia or Zimbabwe or "Central Africa" or "West Africa" or "Western Africa" or "East Africa" or "Eastern Africa" or "North Africa" or "Northern Africa" or "Southern Africa" or "sub Saharan Africa" or "subsaharan Africa" or Africa).ti,ab. 489423 7 4 or 5 2272796 8 (prevalen* or incidence* or burden*).ti,ab. or Prevalence/ or Incidence/ 2974749 9 3 and 6 and 7 and 8 823 10 limit 9 to yr="1860 - 2020" 677 11 limit 9 to yr="2021" 110 | ID Search Hits #1 ((tuberculosis or TB or PTB)):ti,ab,kw (Word variations have been searched) 8804 #2 MeSH descriptor: [Tuberculosis] explode all trees 2503 #3 #1 OR #2 8811 #4 (((hypertension or diabetes or pre-diabetes or cardiovascular or metabolic syndrome or hyperlipid?emia* or dyslipid?emia* or cholesterol or hypercholesterolemia or triglycerides or low density lipoprotein) and (risk* or alcohol or smoking or smoke* or cigarette* or overweight or obese or obesity or physical inactivity))):ti,ab,kw (Word variations have been searched) 92757 #5 MeSH descriptor: [Hypertension] explode all trees 19788 #6 MeSH descriptor: [Diabetes Mellitus] explode all trees 35175 #7 MeSH descriptor: [Obesity] explode all trees 15762 #8 MeSH descriptor: [Heart Disease Risk Factors] explode all trees 305 #9 MeSH descriptor: [Hyperlipidemias] explode all trees 6683 #10 MeSH descriptor: [Metabolic Syndrome] explode all trees 2067 #11 MeSH descriptor: [Dyslipidemias] explode all trees 7918 #12 MeSH descriptor: [Alcohol Drinking] explode all trees 4247 #13 MeSH descriptor: [Smoke] explode all trees 437 #14 #4 OR #5 OR #6 OR #7 OR #8 OR #9 OR #10 OR #11 OR #12 OR #13 147542 #15 ((Algeria or Angola or Benin or Botswana or "Burkina Faso" or Burundi or Cameroon or "Cape Verde" or "Central African Republic" or Chad or Comoros or "Democratic Republic of Congo" or Djibouti or Egypt or "Equatorial Guinea" or Eritrea or Ethiopia or Gabon or Gambia or Ghana or Guinea or "Guinea Bissau" or "Ivory Coast" or "Cote d’Ivoire" or Kenya or Lesotho or Liberia or Libya or Libya or Madagascar or Malawi or Mali or Mauritania or Mauritius or Morocco or Mozambique or Namibia or Niger or Nigeria or Rwanda or "Sao Tome" or Senegal or Seychelles or "Sierra Leone" or Somalia or "South Africa" or "South Sudan" or Sudan or Swaziland or Tanzania or Togo or Tunisia or Uganda or Zaire or Zambia or Zimbabwe or "Central Africa" or "West Africa" or "Western Africa" or "East Africa" or "Eastern Africa" or "North Africa" or "Northern Africa" or "Southern Africa" or "sub Saharan Africa" or "subsaharan Africa" or Africa)):ti,ab,kw (Word variations have been searched) 23650 #16 (prevalen* or incidence* or burden*):ti,ab,kw (Word variations have been searched) 196824 #17 MeSH descriptor: [Prevalence] explode all trees 4952 #18 MeSH descriptor: [Incidence] explode all trees 10734 #19 #16 OR #17 OR #18 196824 #20 #3 AND #14 AND #15 AND #19 22 | 1 (TI=((tuberculosis or TB or PTB))) OR AB=((tuberculosis or TB or PTB)) 2 (TI=(((hypertension or diabetes or pre-diabetes or cardiovascular or metabolic syndrome or hyperlipid?emia* or dyslipid?emia* or cholesterol or hypercholesterolemia or triglycerides or low density lipoprotein) and (risk* or alcohol or smoking or smoke* or cigarette* or overweight or obese or obesity or physical inactivity)))) OR AB=(((hypertension or diabetes or pre-diabetes or cardiovascular or metabolic syndrome or hyperlipid?emia* or dyslipid?emia* or cholesterol or hypercholesterolemia or triglycerides or low density lipoprotein) and (risk* or alcohol or smoking or smoke* or cigarette* or overweight or obese or obesity or physical inactivity))) 3 (TI=((Algeria or Angola or Benin or Botswana or "Burkina Faso" or Burundi or Cameroon or "Cape Verde" or "Central African Republic" or Chad or Comoros or "Democratic Republic of Congo" or Djibouti or Egypt or "Equatorial Guinea" or Eritrea or Ethiopia or Gabon or Gambia or Ghana or Guinea or "Guinea Bissau" or "Ivory Coast" or "Cote d’Ivoire" or Kenya or Lesotho or Liberia or Libya or Libya or Madagascar or Malawi or Mali or Mauritania or Mauritius or Morocco or Mozambique or Namibia or Niger or Nigeria or Rwanda or "Sao Tome" or Senegal or Seychelles or "Sierra Leone" or Somalia or "South Africa" or "South Sudan" or Sudan or Swaziland or Tanzania or Togo or Tunisia or Uganda or Zaire or Zambia or Zimbabwe or "Central Africa" or "West Africa" or "Western Africa" or "East Africa" or "Eastern Africa" or "North Africa" or "Northern Africa" or "Southern Africa" or "sub Saharan Africa" or "subsaharan Africa" or Africa))) OR AB=((Algeria or Angola or Benin or Botswana or "Burkina Faso" or Burundi or Cameroon or "Cape Verde" or "Central African Republic" or Chad or Comoros or "Democratic Republic of Congo" or Djibouti or Egypt or "Equatorial Guinea" or Eritrea or Ethiopia or Gabon or Gambia or Ghana or Guinea or "Guinea Bissau" or "Ivory Coast" or "Cote d’Ivoire" or Kenya or Lesotho or Liberia or Libya or Libya or Madagascar or Malawi or Mali or Mauritania or Mauritius or Morocco or Mozambique or Namibia or Niger or Nigeria or Rwanda or "Sao Tome" or Senegal or Seychelles or "Sierra Leone" or Somalia or "South Africa" or "South Sudan" or Sudan or Swaziland or Tanzania or Togo or Tunisia or Uganda or Zaire or Zambia or Zimbabwe or "Central Africa" or "West Africa" or "Western Africa" or "East Africa" or "Eastern Africa" or "North Africa" or "Northern Africa" or "Southern Africa" or "sub Saharan Africa" or "subsaharan Africa" or Africa)) 4 (TI=((prevalen* or incidence* or burden*))) OR AB=((prevalen* or incidence* or burden*)) |
| **Results: inception - 2020** | 244 | 677 | 17 | 124 |
| **Results: 2021** | 40 | 110 | 5 | 21 |

# REFERENCES

1. Sitas F, Urban M, Bradshaw D, Kielkowski D, Bah S, Peto R. Tobacco attributable deaths in South Africa. *Tob. Control* 2004; 13: 396–399.

2. Sonnenberg P, Murray J, Glynn, Thomas RG, Godfrey-Faussett P, Shearer S. Risk factors for pulmonary disease due to culture-positive M. tuberculosis or nontuberculous mycobacteria in South African gold miners. *Eur. Respir. J.* European Respiratory Society; 2000; 15: 291–296.

3. Iradukunda A, Ndayishimiye G-P, Sinarinzi D, Odjidja EN, Ntakaburimvo N, Nshimirimana I, Izere C. Key factors influencing multidrug-resistant tuberculosis in patients under anti-tuberculosis treatment in two centres in Burundi: a mixed effect modelling study. *BMC Public Health* 2021; 21: 2142.

4. Wekunda PW, Aduda DSO, Guyah B. Determinants of tuberculosis treatment interruption among patients in Vihiga County, Kenya. *PLOS ONE* Public Library of Science; 2021; 16: e0260669.

5. Petersen MR, Nonyane BAS, Lebina L, Mmolawa L, Siwelana T, Martinson N, Dowdy DW, Hanrahan CF. Geographic mobility and time to seeking care among people with TB in Limpopo, South Africa. *Int. J. Tuberc. Lung Dis.* 2021; 25: 708–715.

6. Nyasulu P, Mogoere S, Umanah T, Setswe G. Determinants of Pulmonary Tuberculosis among Inmates at Mangaung Maximum Correctional Facility in Bloemfontein, South Africa. *Tuberc. Res. Treat.* Hindawi; 2015; 2015: e752709.

7. Appiah PK, Osei B, Amu H. Factors associated with nutritional status, knowledge and attitudes among tuberculosis patients receiving treatment in Ghana: A cross-sectional study in the Tema Metropolis. *Plos One* Public Library of Science San Francisco, CA USA; 2021; 16: e0258033.

8. Hill PC, Jackson-Sillah D, Donkor SA, Otu J, Adegbola RA, Lienhardt C. Risk factors for pulmonary tuberculosis: a clinic-based case control study in The Gambia. *BMC Public Health* 2006; 6: 156.

9. Boillat-Blanco N, Ramaiya KL, Mganga M, Minja LT, Bovet P, Schindler C, Von Eckardstein A, Gagneux S, Daubenberger C, Reither K, Probst-Hensch N. Transient Hyperglycemia in Patients With Tuberculosis in Tanzania: Implications for Diabetes Screening Algorithms. *J. Infect. Dis.* 2016; 213: 1163–1172.

10. Ali MH, Alrasheedy AA, Hassali MA, Kibuule D, Godman B. Predictors of Multidrug-Resistant Tuberculosis (MDR-TB) in Sudan. *Antibiotics* [Internet] 2019 [cited 2020 Jan 23]; 8Available from: https://www.ncbi.nlm.nih.gov/pmc/articles/PMC6783989/.

11. Murrison LB, Martinson N, Moloney RM, Msandiwa R, Mashabela M, Samet JM, Golub JE. Tobacco Smoking and Tuberculosis among Men Living with HIV in Johannesburg, South Africa: A Case-Control Study. *PLOS ONE* Public Library of Science; 2016; 11: e0167133.

12. Racil H, Mami M, Chabbou A. Predictive factors for recurrence of pulmonary tuberculosis in Tunisia: a retrospective study. *Rev. Mal. Respir.* 2012; 29: 412–418.

13. Deetlefs E, Epstein D, Watermeyer GA, Seggie RM, Thomson SR. Tuberculosis in an inflammatory bowel disease cohort from South Africa. *S. Afr. Med. J.* 2012; 102: 802–804.

14. Kubjane M, Berkowitz N, Goliath R, Levitt NS, Wilkinson RJ, Oni T. Tuberculosis, Human Immunodeficiency Virus, and the Association With Transient Hyperglycemia in Periurban South Africa. *Clin. Infect. Dis.* 2020; 71: 1080–1088.

15. Magee MJ, Sun YV, Brust JCM, Shah NS, Ning Y, Allana S, Campbell A, Hui Q, Mlisana K, Moodley P, Gandhi NR. Polymorphisms in the vitamin D receptor gene are associated with reduced rate of sputum culture conversion in multidrug-resistant tuberculosis patients in South Africa. *PLoS ONE* [Internet] 2017 [cited 2021 Jun 16]; 12Available from: https://www.ncbi.nlm.nih.gov/pmc/articles/PMC5507304/.

16. Otwombe KN, Variava E, Holmes CB, Chaisson RE, Martinson N. Predictors of delay in the diagnosis and treatment of suspected tuberculosis in HIV co-infected patients in South Africa. *Int. J. Tuberc. Lung Dis.* 2013; 17: 1199–1205.

17. Iweama CN, Agbaje OS, Umoke PCI, Igbokwe CC, Ozoemena EL, Omaka-Amari NL, Idache BM. Nonadherence to tuberculosis treatment and associated factors among patients using directly observed treatment short-course in north-west Nigeria: A cross-sectional study. *SAGE Open Med.* SAGE Publications Ltd; 2021; 9: 2050312121989497.

18. Wotale TW, Terefe AN, Fufa JA. Modeling Time to Death of Patients with Multidrug-Resistant Tuberculosis at Saint Peter’s Specialized Hospital. *J. Res. Health Sci.* 2021; 21: e00513.

19. Tulu B, Amsalu E, Zenebe Y, Abebe M, Fetene Y, Agegn M, Abate A, Ponpetch K, Bekana T, Gumi B, Ameni G. Diabetes mellitus and HIV infection among active tuberculosis patients in Northwest Ethiopia: health facility-based cross-sectional study. *Trop. Med. Health* 2021; 49: 68.

20. Assal GME, AbdelFattah EB, Nabil MM. Characteristics and outcome determinants in patients with pulmonary tuberculosis in ICU. *Egypt. J. Chest Dis. Tuberc.* 2021; 70: 89.

21. Zetola NM, Moonan PK, Click E, Oeltmann JE, Basotli J, Wen X-J, Boyd R, Tobias JL, Finlay A, Modongo C. Population-based geospatial and molecular epidemiologic study of tuberculosis transmission dynamics, Botswana, 2012–2016. *Emerg. Infect. Dis.* Centers for Disease Control and Prevention; 2021; 27: 835.

22. Mitrani L, Dickson-Hall L, Le Roux S, Hill J, Loveday M, Grant AD, Kielmann K, Mlisana K, Moshabela M, Nicol MP, Black J, Cox H. Diverse clinical and social circumstances: developing patient-centred care for DR-TB patients in South Africa. *Public Health Action* 2021; 11: 120–125.

23. Seegert AB, Patsche CB, Sifna A, Gomes VF, Wejse C, Storgaard M, Rudolf F. Hypertension is associated with increased mortality in patients with tuberculosis in Guinea-Bissau. *Int. J. Infect. Dis.* 2021; 109: 123–128.

24. Sidamo T, Shibeshi W, Yimer G, Aklillu E, Engidawork E. Explorative Analysis of Treatment Outcomes of Levofloxacin-and Moxifloxacin-Based Regimens and Outcome Predictors in Ethiopian MDR-TB Patients: A Prospective Observational Cohort Study. *Infect. Drug Resist.* Dove Press; 2021; 14: 5473.

25. Isralls S, Baisley K, Ngam E, Grant AD, Millard J. QT Interval Prolongation in People Treated With Bedaquiline for Drug-Resistant Tuberculosis Under Programmatic Conditions: A Retrospective Cohort Study. *Open Forum Infect. Dis.* 2021; 8: ofab413.

26. Balkissou A, Pefura-Yone E, LM EM, Ngono O, SA AM, Kanko N, Fodjeu G, Fogang D, Kuaban C. Residual pleural opacity in patients treated for pleural tuberculosis in Yaounde. *Rev. Pneumol. Clin.* 2015; 72: 115–121.

27. Whitehouse ER, Perrin N, Levitt N, Hill M, Farley JE. Cardiovascular risk prevalence in South Africans with drug-resistant tuberculosis: a cross-sectional study. *Int. J. Tuberc. Lung Dis. Off. J. Int. Union Tuberc. Lung Dis.* 2019; 23: 587–593.

28. Yorke E, Boima V, Dey ID, Atiase Y, Akpalu J, Yawson AE, Ganu V, Forson A, Mate-Kole CC. Examination of Dysglycaemia among Newly Diagnosed Tuberculosis Patients in Ghana: A Cross-Sectional Study. *Tuberc. Res. Treat.* Hindawi; 2018; 2018: e1830372.

29. Gebrehiwet GB, Kahsay AG, Welekidan LN, Hagos AK, Abay GK, Hagos DG. Rifampicin resistant tuberculosis in presumptive pulmonary tuberculosis cases in Dubti Hospital, Afar, Ethiopia. *J. Infect. Dev. Ctries.* 2019; 13: 21–27.

30. Ayeni FA, Oyetunde OO, Aina BA. The effect of collaborative care on treatment outcomes of newly diagnosed tuberculosis patients with Type-2 diabetes mellitus and adverse drug reaction presentations: A prospective study. *Int. J. Mycobacteriology* 2021; 10: 285–292.

31. Farghaly S, El-Abdeen AZ, Shaaban LH, Mahmoud A. Epidemiology and outcome of rifampicin resistant tuberculosis in Upper Egypt. *Egypt. J. Chest Dis. Tuberc.* Medknow Publications; 2021; 70: 183.

32. Baik Y, Rickman HM, Hanrahan CF, Mmolawa L, Kitonsa PJ, Sewelana T, Nalutaaya A, Kendall EA, Lebina L, Martinson N, Katamba A, Dowdy DW. A clinical score for identifying active tuberculosis while awaiting microbiological results: Development and validation of a multivariable prediction model in sub-Saharan Africa. *PLOS Med.* Public Library of Science; 2020; 17: e1003420.

33. Asante-Poku A, Asare P, Baddoo NA, Forson A, Klevor P, Otchere ID, Aboagye SY, Osei-Wusu S, Danso EK, Koram K, Gagneux S, Yeboah-Manu D. TB-diabetes co-morbidity in Ghana: The importance of Mycobacterium africanum infection. *PLOS ONE* Public Library of Science; 2019; 14: e0211822.

34. Mabula PL, Kazinyingia KI, Chavala EC, Mosha V, Msuya SE, Leyaro BJ. Prevalence and risk factors for diabetes mellitus among tuberculosis patients in Moshi Municipal Council, Kilimanjaro Tanzania. *East Afr. Health Res. J.* 2021; 5: 69–74.

35. Tiamiyu AB, Iliyasu G, Dayyab FM, Habib ZG, Tambuwal SH, Animashaun AO, Galadanci H, Bwala SA, Lawson L, Habib AG. A descriptive study of smear negative pulmonary tuberculosis in a high HIV burden patient’s population in North Central Nigeria. *PLOS ONE* Public Library of Science; 2020; 15: e0238007.

36. Hoog AH van’t, Marston BJ, Ayisi JG, Agaya JA, Muhenje O, Odeny LO, Hongo J, Laserson KF, Borgdorff MW. Risk Factors for Inadequate TB Case Finding in Rural Western Kenya: A Comparison of Actively and Passively Identified TB Patients. *PLOS ONE* Public Library of Science; 2013; 8: e61162.

37. Musuenge BB, Poda GG, Chen P-C. Nutritional Status of Patients with Tuberculosis and Associated Factors in the Health Centre Region of Burkina Faso. *Nutrients* Multidisciplinary Digital Publishing Institute; 2020; 12: 2540.

38. Kirenga BJ, Ssengooba W, Muwonge C, Nakiyingi L, Kyaligonza S, Kasozi S, Mugabe F, Boeree M, Joloba M, Okwera A. Tuberculosis risk factors among tuberculosis patients in Kampala, Uganda: implications for tuberculosis control. *BMC Public Health* [Internet] 2015 [cited 2020 Jun 22]; 15Available from: https://www.ncbi.nlm.nih.gov/pmc/articles/PMC4311451/.

39. Adegbite BR, Edoa JR, Agbo PA, Dejon-Agobé JC, Essone PN, Lotola-Mougeni F, Ngwese MM, Mfoumbi A, Mevyann C, Epola M. Epidemiological, Mycobacteriological, and Clinical Characteristics of Smoking Pulmonary Tuberculosis Patients, in Lambaréné, Gabon: A Cross-Sectional Study. *Am. J. Trop. Med. Hyg.* ASTMH; 2020; 103: 2501–2505.

40. Lam C, Martinson N, Hepp L, Ambrose B, Msandiwa R, Wong ML, Apelberg B, Tamplin S, Golub JE. Prevalence of tobacco smoking in adults with tuberculosis in South Africa. *Int. J. Tuberc. Lung Dis. Off. J. Int. Union Tuberc. Lung Dis.* 2013; 17: 1354–1357.

41. Ugarte-Gil C, Alisjahbana B, Ronacher K, Riza AL, Koesoemadinata RC, Malherbe ST, Cioboata R, Llontop JC, Kleynhans L, Lopez S, Santoso P, Marius C, Villaizan K, Ruslami R, Walzl G, Panduru NM, Dockrell HM, Hill PC, Mc Allister S, Pearson F, Moore DAJ, Critchley JA, van Crevel R, for the TANDEM Consortium. Diabetes Mellitus Among Pulmonary Tuberculosis Patients From 4 Tuberculosis-endemic Countries: The TANDEM Study. *Clin. Infect. Dis.* 2020; 70: 780–788.

42. Faurholt-Jepsen D, Range N, PrayGod G, Jeremiah K, Faurholt-Jepsen M, Aabye MG, Changalucha J, Christensen DL, Witte DR, Andersen AB, Friis H. The role of anthropometric and other predictors for diabetes among urban Tanzanians with tuberculosis. *Int. J. Tuberc. Lung Dis.* 2012; 16: 1680–1685.

43. Kibirige D, Ssekitoleko R, Mutebi E, Worodria W. Overt diabetes mellitus among newly diagnosed Ugandan tuberculosis patients: a cross sectional study. *BMC Infect. Dis.* 2013; 13: 122.

44. Pizzol D, Di Gennaro F, Chhaganlal KD, Fabrizio C, Monno L, Putoto G, Saracino A. Prevalence of diabetes mellitus in newly diagnosed pulmonary tuberculosis in Beira, Mozambique. *Afr. Health Sci.* 2017; 17: 773–779.

45. Mollel EW, Chilongola JO. Predictors for Mortality among Multidrug-Resistant Tuberculosis Patients in Tanzania [Internet]. J. Trop. Med. Hindawi; 2017 [cited 2020 Apr 18]. p. e9241238Available from: https://www.hindawi.com/journals/jtm/2017/9241238/.

46. Kassa GM, Merid MW, Muluneh AG. Khat Chewing and Clinical Conditions Determine the Epidemiology of Primary Drug Resistance Tuberculosis in Amhara Region of Ethiopia: A Multicenter Study. *Infect. Drug Resist.* 2021; 14: 2449–2460.

47. Louwagie GM, Ayo-Yusuf OA. Tobacco use patterns in tuberculosis patients with high rates of human immunodeficiency virus co-infection in South Africa. *BMC Public Health* BioMed Central; 2013; 13: 1–10.

48. Mulisa G, Workneh T, Hordofa N, Suaudi M, Abebe G, Jarso G. Multidrug-resistant Mycobacterium tuberculosis and associated risk factors in Oromia Region of Ethiopia. *Int. J. Infect. Dis.* 2015; 39: 57–61.

49. Segafredo G, Kapur A, Robbiati C, Joseph N, Sousa JR de, Putoto G, Manenti F, Atzori A, Fedeli U. Integrating TB and non-communicable diseases services: Pilot experience of screening for diabetes and hypertension in patients with Tuberculosis in Luanda, Angola. *PLOS ONE* Public Library of Science; 2019; 14: e0218052.

50. Tola HH, Shojaeizadeh D, Garmaroudi G, Tol A, Yekaninejad MS, Ejeta LT, Kebede A, Karimi M, Kassa D. Psychological distress and its effect on tuberculosis treatment outcomes in Ethiopia. *Glob. Health Action* [Internet] 2015 [cited 2021 Jun 20]; 8Available from: https://www.ncbi.nlm.nih.gov/pmc/articles/PMC4660932/.

51. Adetifa IMO, Kendall L, Donkor S, Lugos MD, Hammond AS, Owiafe PK, Ota MOC, Brookes RH, Hill PC. Mycobacterium tuberculosis Infection in Close Childhood Contacts of Adults with Pulmonary Tuberculosis is Increased by Secondhand Exposure to Tobacco. *Am. J. Trop. Med. Hyg.* The American Society of Tropical Medicine and Hygiene; 2017; 97: 429–432.

52. Shangase Z, Tsoka-Gwegweni JM, Okem A. Smoking prevalence among inpatients with drug resistant tuberculosis in KwaZulu-Natal, South Africa. *Tob. Induc. Dis.* The International Society for the Prevention of Tobacco Induced Diseases; 2018; 16.

53. Oni T, Berkowitz N, Kubjane M, Goliath R, Levitt NS, Wilkinson RJ. Trilateral overlap of tuberculosis, diabetes and HIV-1 in a high-burden African setting: implications for TB control. *Eur. Respir. J.* [Internet] European Respiratory Society; 2017 [cited 2021 Jun 25]; 50Available from: https://erj.ersjournals.com/content/50/1/1700004.

54. Nagu T, Ray R, Munseri P, Moshiro C, Shayo G, Kazema R, Mugusi F, Pallangyo K. Tuberculosis among the elderly in Tanzania: disease presentation and initial response to treatment. *Int. J. Tuberc. Lung Dis.* International Union Against Tuberculosis and Lung Disease; 2017; 21: 1251–1257.

55. Haraldsdottir TL, Rudolf F, Bjerregaard-Andersen M, Carlos Joaquím L, Stochholm K, Gomes VF, Beck-Nielsen H, Ostergaard L, Aaby P, Wejse C. Diabetes mellitus prevalence in tuberculosis patients and the background population in Guinea-Bissau: a disease burden study from the capital Bissau. *Trans. R. Soc. Trop. Med. Hyg.* 2015; 109: 400–407.

56. Kufa T, Chihota V, Mngomezulu V, Charalambous S, Verver S, Churchyard G, Borgdorff M. The incidence of tuberculosis among hiv-positive individuals with high CD4 counts: implications for policy. *BMC Infect. Dis.* 2016; 16: 266.

57. Dalton T, Cegielski P, Akksilp S, Asencios L, Caoili JC, Cho S-N, Erokhin VV, Ershova J, Gler MT, Kazennyy BY. Prevalence of and risk factors for resistance to second-line drugs in people with multidrug-resistant tuberculosis in eight countries: a prospective cohort study. *The Lancet* Elsevier; 2012; 380: 1406–1417.

58. Sattar S, Van Schalkwyk C, Claassens M, Dunbar R, Floyd S, Enarson DA, Godfrey-Faussett P, Ayles H, Beyers N. Symptom reporting among prevalent tuberculosis cases who smoke, are HIV-positive or have hyperglycaemia. *Public Health Action* 2014; 4: 222–225.

59. Molalign S, Wencheko E. Risk factors of mortality in patients with multi-drug resistant TB. *Ethiop. J. Health Dev.* [Internet] 2015 [cited 2021 Jun 15]; 29Available from: https://ejhd.org/index.php/ejhd/article/view/147.

60. Fwoloshi S, Hachaambwa LM, Chiyeñu KO, Chirwa L, Hoffman TW, Ngalamika O, Bailey SL. Screening for Diabetes Mellitus among Tuberculosis Patients: Findings from a Study at a Tertiary Hospital in Lusaka, Zambia. *Can. J. Infect. Dis. Med. Microbiol.* Hindawi; 2018; 2018: e3524926.

61. Diandé S, Badoum G, Combary A, Zombra I, Saouadogo T, Sawadogo LT, Nébié B, Gnanou S, Zigani A, Ouédraogo SM, Diallo A, Kaboré S, Sangaré L. Multidrug-Resistant Tuberculosis in Burkina Faso from 2006 to 2017: Results of National Surveys. *Eur. J. Microbiol. Immunol.* 2019; 9: 23–28.

62. Ade S, Affolabi D, Agodokpessi G, Wachinou P, Faïhun F, Toundoh N, Békou W, Makpenon A, Ade G, Anagonou S, Harries AD. Low prevalence of diabetes mellitus in patients with tuberculosis in Cotonou, Benin. *Public Health Action* 2015; 5: 147–149.

63. Mwiru RS, Nagu TJ, Kaduri P, Mugusi F, Fawzi W. Prevalence and patterns of cigarette smoking among patients co-infected with human immunodeficiency virus and tuberculosis in Tanzania. *Drug Alcohol Depend.* 2017; 170: 128–132.

64. Naidoo P, Peltzer K, Louw J, Matseke G, Mchunu G, Tutshana B. Predictors of tuberculosis (TB) and antiretroviral (ARV) medication non-adherence in public primary care patients in South Africa: a cross sectional study. *BMC Public Health* Springer; 2013; 13: 1–10.

65. Munseri PJ, Kimambo H, Pallangyo K. Diabetes mellitus among patients attending TB clinics in Dar es Salaam: a descriptive cross-sectional study. *BMC Infect. Dis.* BioMed Central; 2019; 19: 1–8.

66. Stracker N, Hanrahan C, Mmolawa L, Nonyane B, Tampi R, Tucker A, West N, Lebina L, Martinson N, Dowdy D. Risk factors for catastrophic costs associated with tuberculosis in rural South Africa. *Int. J. Tuberc. Lung Dis. Off. J. Int. Union Tuberc. Lung Dis.* 2019; 23: 756–763.

67. Ekeke N, Ukwaja KN, Chukwu JN, Nwafor CC, Meka AO, Egbagbe EE, Soyinka FO, Alobu I, Agujiobi I, Akingbesote S, Igbinigie O, Offor JB, Madichie NO, Alphonsus C, Anyim MC, Mbah OK, Oshi DC. Screening for diabetes mellitus among tuberculosis patients in Southern Nigeria: a multi-centre implementation study under programme settings. *Sci. Rep.* [Internet] 2017 [cited 2021 Jun 15]; 7Available from: https://www.ncbi.nlm.nih.gov/pmc/articles/PMC5345020/.

68. Berkowitz N, Okorie A, Goliath R, Levitt N, Wilkinson RJ, Oni T. The prevalence and determinants of active tuberculosis among diabetes patients in Cape Town, South Africa, a high HIV/TB burden setting. *Diabetes Res. Clin. Pract.* 2018; 138: 16–25.

69. Desta KT, Masango TE, Nkosi ZZ. Performance of the National Tuberculosis Control Program in the post conflict Liberia. *PLoS ONE* 2018; 13: e0199474.

70. Peltzer K, Naidoo P, Matseke G, Louw J, Mchunu G, Tutshana B. Prevalence of post-traumatic stress symptoms and associated factors in tuberculosis (TB), TB retreatment and/or TB–HIV co-infected primary public health-care patients in three districts in South Africa. *Psychol. Health Med.* Taylor & Francis; 2013; 18: 387–397.

71. Ngosa K, Naidoo RN. The risk of pulmonary tuberculosis in underground copper miners in Zambia exposed to respirable silica: a cross-sectional study. *BMC Public Health* 2016; 16: 855.

72. Gunasekera K, Cohen T, Gao W, Ayles H, Godfrey-Faussett P, Claassens M. Smoking and HIV associated with subclinical tuberculosis: analysis of a population-based prevalence survey. *Int. J. Tuberc. Lung Dis.* International Union Against Tuberculosis and Lung Disease; 2020; 24: 340–346.

73. Wessels J, Walsh CM, Nel M. Smoking habits and alcohol use of patients with tuberculosis at Standerton Tuberculosis Specialised Hospital, Mpumalanga, South Africa. *Health SA SA Gesondheid* 2019; 24: 1146.

74. Mburu JW, Kingwara L, Ester M, Andrew N. Prognostic factors among TB and TB/DM comorbidity among patients on short course regimen within Nairobi and Kiambu counties in Kenya. *J. Clin. Tuberc. Mycobact. Dis.* 2018; 12: 9–13.

75. Kombila U, Mbaye F, Ka W, NO TB. Clinical and radiological characteristics of pulmonary tuberculosis in tobacco smokers. *Rev. Mal. Respir.* 2018; 35: 538–545.

76. Brunet L, Pai M, Davids V, Ling D, Paradis G, Lenders L, Meldau R, van Zyl Smit R, Calligaro G, Allwood B, Dawson R, Dheda K. High prevalence of smoking among patients with suspected tuberculosis in South Africa. *Eur. Respir. J.* 2011; 38: 139–146.

77. Azeez A, Ndege J, Mutambayi R. Associated factors with unsuccessful tuberculosis treatment outcomes among tuberculosis/HIV coinfected patients with drug-resistant tuberculosis. *Int. J. Mycobacteriology* Medknow Publications; 2018; 7: 347.

78. Watermeyer G, Thomson S. Differentiating Crohn’s disease from intestinal tuberculosis at presentation in patients with tissue granulomas. *S. Afr. Med. J.* 2018; 108: 399–402.

79. Kootbodien T, Wilson K, Tlotleng N, Ntlebi V, Made F, Rees D, Naicker N. Tuberculosis Mortality by Occupation in South Africa, 2011–2015. *Int. J. Environ. Res. Public. Health* Multidisciplinary Digital Publishing Institute; 2018; 15: 2756.

80. Soboka M, Tesfaye M, Adorjan K, Krahl W, Tesfaye E, Yitayih Y, Strobl R, Grill E. Effect of food insecurity on mental health of patients with tuberculosis in Southwest Ethiopia: a prospective cohort study. *BMJ Open* 2021; 11: e045434.

81. Dayyab FM, Iliyasu G, Ahmad BG, Habib AG. Early safety and efficacy of linezolid-based combination therapy among patients with drug-resistant tuberculosis in North-western Nigeria. *Int. J. Mycobacteriology* 2021; 10: 129–135.

82. Soboka M, Tesfaye M, Adorjan K, Krahl W, Tesfaye E, Yitayih Y, Strobl R, Grill E. Substance use disorders and adherence to antituberculosis medications in Southwest Ethiopia: a prospective cohort study. *BMJ Open* British Medical Journal Publishing Group; 2021; 11: e043050.

83. Oumer N, Atnafu DD, Worku GT, Tsehay AK. Determinants of Multi-drug resistant Tuberculosis in four treatment centers of Eastern Amhara, Ethiopia: A case-control study. *J. Infect. Dev. Ctries.* 2021; 15: 687–695.

84. Zetola NM, Modongo C, Kip EC, Gross R, Bisson GP, Collman RG. Alcohol use and abuse among patients with multidrug-resistant tuberculosis in Botswana. *Int. J. Tuberc. Lung Dis. Off. J. Int. Union Tuberc. Lung Dis.* 2012; 16: 1529–1534.

85. Peltzer K, Naidoo P, Louw J, Matseke G, Zuma K, Mchunu G, Tutshana B, Mabaso M. Screening and brief interventions for hazardous and harmful alcohol use among patients with active tuberculosis attending primary public care clinics in South Africa: results from a cluster randomized controlled trial. *BMC Public Health* 2013; 13: 699.

86. Churchyard GJ, Fielding KL, Lewis JJ, Chihota VN, Hanifa Y, Grant AD. Symptom and chest radiographic screening for infectious tuberculosis prior to starting isoniazid preventive therapy: yield and proportion missed at screening. *AIDS* 2010; 24: S19.

87. Bhana A, Rathod SD, Selohilwe O, Kathree T, Petersen I. Characteristics and correlates of alcohol consumption among adult chronic care patients in North West Province, South Africa. *South Afr. Med. J. Suid-Afr. Tydskr. Vir Geneeskd.* 2017; 107: 636–642.

88. Wanyonyi AW, Wanjala PM, Githuku J, Oyugi E, Kutima H. Factors associated with interruption of tuberculosis treatment among patients in Nandi County, Kenya 2015. *Pan Afr. Med. J.* 2017; 28: 11.

89. Hayes-Larson E, Hirsch-Moverman Y, Saito S, Frederix K, Pitt B, Maama-Maime L, Howard A. Depressive symptoms and hazardous/harmful alcohol use are prevalent and correlate with stigma among TB-HIV patients in Lesotho. *Int. J. Tuberc. Lung Dis.* International Union Against Tuberculosis and Lung Disease; 2017; 21: S34–S41.

90. O’Connell R, Chishinga N, Kinyanda E, Patel V, Ayles H, Weiss HA, Seedat S. Prevalence and correlates of alcohol dependence disorder among TB and HIV infected patients in Zambia. *PloS One* Public Library of Science; 2013; 8: e74406.

91. Matseke G, Peltzer K, Louw J, Naidoo P, Mchunu G, Tutshana B. Inconsistent condom use among public primary care patients with tuberculosis in South Africa. *Sci. World J.* Hindawi; 2012; 2012.

92. Muture BN, Keraka MN, Kimuu PK, Kabiru EW, Ombeka VO, Oguya F. Factors associated with default from treatment among tuberculosis patients in nairobi province, Kenya: A case control study. *BMC Public Health* 2011; 11: 696.

93. Talbot EA, Kenyon TA, Moeti TL, Hsin G, Dooley L, El-Halabi S, Binkin NJ. HIV risk factors among patients with tuberculosis—Botswana 1999. *Int. J. STD AIDS* SAGE Publications Sage UK: London, England; 2002; 13: 311–317.

94. Conradie F, Mabiletsa T, Sefoka M, Mabaso S, Louw R, Evans D, Van Rie A. Prevalence and incidence of symmetrical symptomatic peripheral neuropathy in patients with multidrugresistant TB. *S. Afr. Med. J.* 2014; 104: 24–26.

95. Kebede W, Gudina EK, Balay G, Abebe G. Diagnostic implications and inpatient mortality related to tuberculosis at Jimma Medical Center, southwest Ethiopia. *J. Clin. Tuberc. Mycobact. Dis.* 2021; 23: 100220.

96. Kirubi B, Ong’ang’o J, Nguhiu P, Lönnroth K, Rono A, Sidney-Annerstedt K. Determinants of household catastrophic costs for drug sensitive tuberculosis patients in Kenya. *Infect. Dis. Poverty* 2021; 10: 95.

97. Pillay S, Magula NP. Treatment outcomes of Gene Xpert positive tuberculosis patients in KwaMashu Community Health Centre, KwaZulu-Natal, South Africa: A retrospective review. *South. Afr. J. Infect. Dis.* 2021; 36: 217.

98. Erisa KC, Robsky KO, Kitonsa PJ, Nalutaaya A, Isooba D, Nakasolya O, Mukiibi J, Dowdy D, Kendall EA, Katamba A. Low prevalence of diabetes mellitus in TB patients and the community in urban Uganda. *Int. J. Tuberc. Lung Dis. Off. J. Int. Union Tuberc. Lung Dis.* 2021; 25: 590–592.

99. Ncube RT, Dube SA, Machekera SM, Timire C, Zishiri C, Charambira K, Mapuranga T, Duri C, Sandy C, Dlodlo RA, Lin Y. Feasibility and yield of screening for diabetes mellitus among tuberculosis patients in Harare, Zimbabwe. *Public Health Action* 2019; 9: 72–77.

100. Nsonga J, Dongo JP, Mugabe F, Mutungi G, Walyomo R, Oundo C, Zalwango S, Okello D, Muchuro S, Dlodlo RA, Lin Y. Screening tuberculosis patients for diabetes mellitus in a routine program setting in Kampala, Uganda: a cross-sectional study. *F1000Research* 2019; 8: 872.

101. Peltzer K. Tuberculosis non-communicable disease comorbidity and multimorbidity in public primary care patients in South Africa. *Afr. J. Prim. Health Care Fam. Med.* [Internet] 2018 [cited 2021 Jun 15]; 10Available from: https://www.ncbi.nlm.nih.gov/pmc/articles/PMC5913762/.

102. Mcebula V, Crowther NJ, Nagel SE, George JA. Diabetes and abnormal glucose tolerance in subjects with tuberculosis in a South African urban center. *Int. J. Tuberc. Lung Dis.* 2017; 21: 208–213.

103. Owiti P, Keter A, Harries A, Pastakia S, Wambugu C, Kirui N, Kasera G, Momanyi R, Masini E, Some F. Diabetes and pre-diabetes in tuberculosis patients in western Kenya using point-of-care glycated haemoglobin. *Public Health Action* International Union Against Tuberculosis and Lung Disease; 2017; 7: 147–154.

104. Lawson L, Muc M, Oladimeji O, Iweha C, Opoola B, Abdurhaman ST, Bimba JS, Cuevas LE. Tuberculosis and diabetes in Nigerian patients with and without HIV. *Int. J. Infect. Dis.* Elsevier; 2017; 61: 121–125.

105. Umanah T, Ncayiyana J, Padanilam X, Nyasulu PS. Treatment outcomes in multidrug resistant tuberculosis-human immunodeficiency virus Co-infected patients on anti-retroviral therapy at Sizwe Tropical Disease Hospital Johannesburg, South Africa. *BMC Infect. Dis.* 2015; 15: 478.

106. Oni T, Youngblood E, Boulle A, McGrath N, Wilkinson RJ, Levitt NS. Patterns of HIV, TB, and non-communicable disease multi-morbidity in peri-urban South Africa- a cross sectional study. *BMC Infect. Dis.* 2015; 15: 20.

107. Faurholt-Jepsen D, Range N, PrayGod G, Jeremiah K, Faurholt-Jepsen M, Aabye MG, Changalucha J, Christensen DL, Pipper CB, Krarup H, Witte DR, Andersen AB, Friis H. Diabetes Is a Risk Factor for Pulmonary Tuberculosis: A Case-Control Study from Mwanza, Tanzania. *PLOS ONE* Public Library of Science; 2011; 6: e24215.

108. Araia ZZ, Mesfin AB, Mebrahtu AH, Tewelde AG, Osman R, Tuumzghi HA. Diabetes mellitus and its associated factors in tuberculosis patients in maekel region, eritrea: analytical cross-sectional study. *Diabetes Metab. Syndr. Obes. Targets Ther.* Dove Press; 2021; 14: 515.

109. Huis in ’t Veld D, Pengpid S, Colebunders R, Peltzer K. Body Mass Index and Waist Circumference in Patients with HIV in South Africa and Associated Socio-demographic, Health Related and Psychosocial Factors. *AIDS Behav.* 2018; 22: 1972–1986.

110. Bailey SL, Ayles H, Beyers N, Godfrey-Faussett P, Muyoyeta M, du Toit E, Yudkin JS, Floyd S. The association of hyperglycaemia with prevalent tuberculosis: a population-based cross-sectional study. *BMC Infect. Dis.* 2016; 16: 733.

111. Hoy D, Brooks P, Woolf A, Blyth F, March L, Bain C, Baker P, Smith E, Buchbinder R. Assessing risk of bias in prevalence studies: modification of an existing tool and evidence of interrater agreement. *J. Clin. Epidemiol.* Elsevier; 2012; 65: 934–939.

112. Harling G, Ehrlich R, Myer L. The social epidemiology of tuberculosis in South Africa: a multilevel analysis. *Soc. Sci. Med. 1982* 2008; 66: 492–505.

113. Webb EA, Hesseling AC, Schaaf HS, Gie RP, Lombard CJ, Spitaels A, Delport S, Marais BJ, Donald K, Hindmarsh P, Beyers N. High prevalence of Mycobacterium tuberculosis infection and disease in children and adolescents with type 1 diabetes mellitus. *Int. J. Tuberc. Lung Dis. Off. J. Int. Union Tuberc. Lung Dis.* 2009; 13: 868–874.

114. Badawi A, Sayegh S, Sallam M, Sadoun E, Al-Thani M, Alam MW, Arora P. The global relationship between the prevalence of diabetes mellitus and incidence of tuberculosis: 2000-2012. *Glob. J. Health Sci.* 2014; 7: 183–191.

115. Philips L, Visser J, Nel D, Blaauw R. The association between tuberculosis and the development of insulin resistance in adults with pulmonary tuberculosis in the Western sub-district of the Cape Metropole region, South Africa: a combined cross-sectional, cohort study. *BMC Infect. Dis.* 2017; 17: 570.

116. Harries AD, Lin Y, Satyanarayana S, Lönnroth K, Li L, Wilson N, Chauhan LS, Zachariah R, Baker MA, Jeon CY, Murray MB, Maher D, Bygbjerg IC, Enarson DA, Billo NE, Kapur A. The looming epidemic of diabetes-associated tuberculosis: learning lessons from HIV-associated tuberculosis. *Int. J. Tuberc. Lung Dis. Off. J. Int. Union Tuberc. Lung Dis.* 2011; 15: 1436–1444, i.

117. Janse Van Rensburg A, Dube A, Curran R, Ambaw F, Murdoch J, Bachmann M, Petersen I, Fairall L. Comorbidities between tuberculosis and common mental disorders: a scoping review of epidemiological patterns and person-centred care interventions from low-to-middle income and BRICS countries. *Infect. Dis. Poverty* 2020; 9: 4.

118. Riza AL, Pearson F, Ugarte-Gil C, Alisjahbana B, van de Vijver S, Panduru NM, Hill PC, Ruslami R, Moore D, Aarnoutse R, Critchley JA, van Crevel R. Clinical management of concurrent diabetes and tuberculosis and the implications for patient services. *Lancet Diabetes Endocrinol.* 2014; 2: 740–753.

119. Renzaho AMN. The post-2015 development agenda for diabetes in sub-Saharan Africa: challenges and future directions. *Glob. Health Action* 2015; 8: 10.3402/gha.v8.27600.

120. Marais BJ, Lönnroth K, Lawn SD, Migliori GB, Mwaba P, Glaziou P, Bates M, Colagiuri R, Zijenah L, Swaminathan S, Memish ZA, Pletschette M, Hoelscher M, Abubakar I, Hasan R, Zafar A, Pantaleo G, Craig G, Kim P, Maeurer M, Schito M, Zumla A. Tuberculosis comorbidity with communicable and non-communicable diseases: integrating health services and control efforts. *Lancet Infect. Dis.* 2013; 13: 436–448.

121. Njuguna B, Vorkoper S, Patel P, Reid MJA, Vedanthan R, Pfaff C, Park PH, Fischer L, Laktabai J, Pastakia SD. Models of integration of HIV and noncommunicable disease care in sub-Saharan Africa: lessons learned and evidence gaps. *AIDS Lond. Engl.* 2018; 32: S33–S42.

122. Germe M, Zingwari J, Matji R, Mbambo B. Baseline Assessment of High Volume Facility Capacity to Provide Integrated Tuberculosis (TB) and Diabetes (DM) Services in South Africa. AMER DIABETES ASSOC 1701 N BEAUREGARD ST, ALEXANDRIA, VA 22311-1717 USA; 2017. p. A460–A461.

123. Bartlett S, Gemiarto AT, Ngo MD, Sajiir H, Hailu S, Sinha R, Foo CX, Kleynhans L, Tshivhula H, Webber T. GPR183 Regulates Interferons, Autophagy, and Bacterial Growth During Mycobacterium tuberculosis Infection and Is Associated With TB Disease Severity. *Front. Immunol.* Frontiers Media SA; 2020; 11.

124. Harries AD, Murray MB, Jeon CY, Ottmani S-E, Lonnroth K, Barreto ML, Billo N, Brostrom R, Bygbjerg IC, Fisher-Hoch S, Mori T, Ramaiya K, Roglic G, Strandgaard H, Unwin N, Viswanathan V, Whiting D, Kapur A. Defining the research agenda to reduce the joint burden of disease from Diabetes mellitus and Tuberculosis. *Trop. Med. Int. Health* 2010; 15: 659–663.

125. Harries AD, Dlodlo RA, Brigden G, Mortimer K, Jensen P, Fujiwara PI, Castro JL, Chakaya JM. Should we consider a ‘fourth 90’ for tuberculosis? *Int. J. Tuberc. Lung Dis.* 2019; 23: 1253–1256.

126. Okunola OO, Akintunde AA, Akinwusi PO. Some emerging issues in medical admission pattern in the tropics. *Niger. J. Clin. Pract.* 2012; 15: 51–54.

127. Young F, Critchley JA, Johnstone LK, Unwin NC. A review of co-morbidity between infectious and chronic disease in Sub Saharan Africa: TB and Diabetes Mellitus, HIV and Metabolic Syndrome, and the impact of globalization. *Glob. Health* 2009; 5: 9.

128. Louw J, Peltzer K, Naidoo P, Matseke G, Mchunu G, Tutshana B. Quality of life among tuberculosis (TB), TB retreatment and/or TB-HIV co-infected primary public health care patients in three districts in South Africa. *Health Qual. Life Outcomes* 2012; 10: 77.

129. Louw JS, Mabaso M, Peltzer K. Change in Health-Related Quality of Life among Pulmonary Tuberculosis Patients at Primary Health Care Settings in South Africa: A Prospective Cohort Study. *PloS One* 2016; 11: e0151892.

130. Peltzer K. Conjoint alcohol and tobacco use among tuberculosis patients in public primary healthcare in South Africa. *South Afr. J. Psychiatry* 2014; 20: 6.

131. Peltzer K. Decline of common mental disorders over time in public primary care tuberculosis patients in South Africa. *Int. J. Psychiatry Med.* 2016; 51: 236–245.

132. Peltzer K, Louw J. Prevalence of suicidal behaviour & associated factors among tuberculosis patients in public primary care in South Africa. *Indian J. Med. Res.* 2013; 138: 194–200.

133. Peltzer K, Louw JS. Prevalence and factors associated with tuberculosis treatment outcome among hazardous or harmful alcohol users in public primary health care in South Africa. *Afr. Health Sci.* 2014; 14: 157–166.

134. Peltzer K, McHunu G, Tutshana B, Naidoo P, Matseke G, Louw J. Predictors of non-uptake of human immunodeficiency virus testing by tuberculosis public primary patients in three districts, South Africa. *Iran. J. Public Health* 2012; 41: 19–26.

135. Peltzer K, Louw J, Mchunu G, Naidoo P, Matseke G, Tutshana B. Hazardous and Harmful Alcohol Use and Associated Factors in Tuberculosis Public Primary Care Patients in South Africa. *Int. J. Environ. Res. Public. Health* 2012; 9: 3245–3257.

136. Peltzer K, Naidoo P, Matseke G, Louw J, McHunu G, Tutshana B. Prevalence of psychological distress and associated factors in tuberculosis patients in public primary care clinics in South Africa. *BMC Psychiatry* 2012; 12: 89.

137. Magu D, Wambui E, Ngure K, Karanja S. Treatment outcome among newly diagnosed tuberculosis patients in Kenya. *Ann. Glob. Health* Ubiquity Press; 2016; 82: 578–579.

138. Wachinou AP, Ade S, Ndour Mbaye M, Bah B, Baldé N, Gninkoun J, Bekou W, Sarr M, Bah Sow O, Affolabi D, Merle C. Tuberculosis prevalence and associated factors among persons with diabetes mellitus after intensified case finding in three West African countries. *Multidiscip. Respir. Med.* 2021; 16: 783.

139. Hopkins KL, Hlongwane KE, Otwombe K, Dietrich J, Jaffer M, Cheyip M, Olivier J, Rooyen H van, Wade AN, Doherty T, Gray GE. Does peer-navigated linkage to care work? A cross-sectional study of active linkage to care within an integrated non-communicable disease-HIV testing centre for adults in Soweto, South Africa. *PLOS ONE* Public Library of Science; 2020; 15: e0241014.

140. Mzembe T, Lessells R, Karat AS, Randera-Rees S, Edwards A, Khan P, Tomita A, Tanser F, Baisley K, Grant AD. Prevalence and Risk Factors for Mycobacterium tuberculosis Infection Among Adolescents in Rural South Africa. *Open Forum Infect. Dis.* 2021; 8: ofaa520.

141. Mohamed SF, Haregu TN, Uthman OA, Khayeka-Wandabwa C, Muthuri SK, Asiki G, Kyobutungi C, Gill P. Multimorbidity from chronic conditions among adults in urban slums: the AWI-Gen Nairobi site study findings. *Glob. Heart* World Heart Federation; 2021; 16.

142. Alisjahbana B, McAllister SM, Ugarte-Gil C, Panduru NM, Ronacher K, Koesoemadinata RC, Zubiate C, Riza AL, Malherbe ST, Kleynhans L, Lopez S, Dockrell HM, Ruslami R, Ioana M, Walzl G, Pearson F, Critchley JA, Moore DAJ, van Crevel R, Hill PC, on behalf of the TANDEM Consortium. Screening diabetes mellitus patients for pulmonary tuberculosis: a multisite study in Indonesia, Peru, Romania and South Africa. *Trans. R. Soc. Trop. Med. Hyg.* 2021; 115: 634–643.

143. Wong EB, Olivier S, Gunda R, Koole O, Surujdeen A, Gareta D, Munatsi D, Modise TH, Dreyer J, Nxumalo S, Smit TK, Ording-Jespersen G, Mpofana IB, Khan K, Sikhosana ZEL, Moodley S, Shen Y-J, Khoza T, Mhlongo N, Bucibo S, Nyamande K, Baisley KJ, Cuadros D, Tanser F, Grant AD, Herbst K, Seeley J, Hanekom WA, Ndung’u T, Siedner MJ, et al. Convergence of infectious and non-communicable disease epidemics in rural South Africa: a cross-sectional, population-based multimorbidity study. *Lancet Glob. Health* Elsevier; 2021; 9: e967–e976.

144. Henry RT, Jiamsakul A, Law M, Losso M, Kamarulzaman A, Phanuphak P, Kumarasamy N, Foulkes S, Mohapi L, Nwizu C, Wood R, Kelleher A, Polizzotto M, SECOND-LINE Study Group. Factors Associated With and Characteristic of HIV/Tuberculosis Co-Infection: A Retrospective Analysis of SECOND-LINE Clinical Trial Participants. *J. Acquir. Immune Defic. Syndr. 1999* 2021; 87: 720–729.
